# Renal Endothelial Cell‐Targeted Extracellular Vesicles Protect the Kidney from Ischemic Injury

**DOI:** 10.1002/advs.202204626

**Published:** 2022-11-23

**Authors:** Kaiyue Zhang, Rongrong Li, Xiaoniao Chen, Hongyu Yan, Huifang Li, Xiaotong Zhao, Haoyan Huang, Shang Chen, Yue Liu, Kai Wang, Zhibo Han, Zhong‐Chao Han, Deling Kong, Xiang‐Mei Chen, Zongjin Li

**Affiliations:** ^1^ School of Medicine Nankai University Tianjin 300071 China; ^2^ The Key Laboratory of Bioactive Materials Ministry of Education College of Life Sciences Nankai University Tianjin 300071 China; ^3^ Beijing Tongren Eye Center Beijing Tongren Hospital Capital Medical University Beijing 100730 China; ^4^ State Key Laboratory of Kidney Diseases Chinese PLA General Hospital Beijing 100853 China; ^5^ Henan Key Laboratory of Medical Tissue Regeneration Xinxiang Medical University Xinxiang Henan 453003 China; ^6^ Jiangxi Engineering Research Center for Stem Cell Shangrao Jiangxi 334000 China; ^7^ Tianjin Key Laboratory of Engineering Technologies for Cell Pharmaceutical National Engineering Research Center of Cell Products AmCellGene Co., Ltd Tianjin 300457 China; ^8^ Beijing Engineering Laboratory of Perinatal Stem Cells Beijing Institute of Health and Stem Cells Health & Biotech Co Beijing 100176 China; ^9^ Tianjin Key Laboratory of Human Development and Reproductive Regulation Tianjin Central Hospital of Gynecology Obstetrics Nankai University Affiliated Hospital of Obstetrics and Gynecology Tianjin 300100 China

**Keywords:** acute kidney injury, endothelial cell, extracellular vesicle, P‐selectin binding peptide, theranostics

## Abstract

Endothelial cell injury plays a critical part in ischemic acute kidney injury (AKI) and participates in the progression of AKI. Targeting renal endothelial cell therapy may ameliorate vascular injury and further improve the prognosis of ischemic AKI. Here, P‐selectin as a biomarker of ischemic AKI in endothelial cells is identified and P‐selectin binding peptide (PBP)‐engineered extracellular vesicles (PBP‐EVs) with imaging and therapeutic functions are developed. The results show that PBP‐EVs exhibit a selective targeting tendency to injured kidneys, while providing spatiotemporal information for the early diagnosis of AKI by quantifying the expression of P‐selectin in the kidneys by molecular imaging. Meanwhile, PBP‐EVs reveal superior nephroprotective functions in the promotion of renal repair and inhibition of fibrosis by alleviating inflammatory infiltration, improving reparative angiogenesis, and ameliorating maladaptive repair of the renal parenchyma. In conclusion, PBP‐EVs, as an ischemic AKI theranostic system that is designed in this study, provide a spatiotemporal diagnosis in the early stages of AKI to help guide personalized therapy and exhibit superior nephroprotective effects, offering proof‐of‐concept data to design EV‐based theranostic strategies to promote renal recovery and further improve long‐term outcomes following AKI.

## Graphical abstract

Endothelial cell (EC) injury is one of the specific features of acute kidney injury (AKI). However, there is no feasible target or effective theranostic strategy for EC‐targeted AKI treatment. In the present study, the authors identify P‐selectin as a biomarker of ischemic AKI and develop P‐selectin binding peptide (PBP)‐engineered extracellular vesicles with imaging and therapeutic functions for the first time.

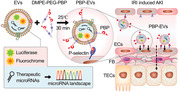

## 1 Introduction

Acute kidney injury (AKI), as a major public health problem associated with high morbidity, can be caused by widespread etiologies, but a large proportion of AKI in the clinic is due to ischemia/reperfusion injury (IRI).^[^ ^1^ ^]^ Vascular injury has been increasingly considered to play a crucial role in the pathophysiology of AKI and further chronic kidney disease (CKD） progression.^[^ ^2^ ^]^ During the early initiation phase of AKI, the insult of endothelial cells (ECs) compromises renal function in several ways by altering capillary integrity, adhering inflammatory cells, and causing vascular rarefaction; any or all of these pathogeneses will prolong the hypoxic state of the kidney and lead to AKI to CKD progression.^[^ ^3^ ^]^

Thus, the development of therapeutic strategies to target injured ECs has enormous potential to protect against AKI and slow the progression of CKD. To this end, a reliable molecular target should be validated first for injured EC‐targeted renal therapy. P‐selectin, as a cell adhesion molecule that is rapidly expressed on the surface of vascular ECs post injury, is responsible for mediating leukocyte slow rolling, arrest, and migration into the injured kidney post AKI.^[^ ^4^ ^]^ Therefore, the severity of renal injury mediated by inflammatory cells is determined primarily by the expression of P‐selectin.^[^ ^5^ ^]^ Blocking P‐selectin on injured ECs has been confirmed to prevent renal injury and rescue kidney function post AKI.^[^ ^6^ ^]^ These pathophysiological processes inspire us to believe that the development of injured EC‐targeted renal therapy using P‐selectin as the target may be a potential strategy to protect against AKI and subsequent progression of CKD.

Extracellular vesicles (EVs), as membrane‐bound natural particles with the ability to transport informational molecules in the form of nucleic acids, proteins, and lipids to mediate intercellular communication, are considered a potential cell‐free therapy for AKI.^[^ ^7^ ^]^ In addition, the unique advantages of EVs, including agent‐loaded space and modifiable properties, make it possible to integrate the targeting ligand, therapeutic ingredient, and imaging agents into one EV and maintain their functionalities simultaneously, which makes EVs excellent candidates as theranostic carriers for EC‐targeted renal therapy.^[^ ^8^ ^]^ Accordingly, a theranostic strategy by tailoring engineered EVs with targeting ligands, therapeutic ingredients, and imaging agents is promising to be developed in which imaging agents are used to provide accurate, visualized, and informative monitoring, thus allowing the early implementation of EC‐targeted therapeutic interventions to promote renal recovery and improve long‐term outcomes following AKI.

Here, we describe the development of a theranostic strategy based on P‐selectin‐targeted EVs with imaging and therapeutic functions for monitoring and targeted treatment of AKI by selective binding to injured ECs. First, we validated the feasibility of P‐selectin as a molecular target for the specific binding of injured ECs in IRI‐induced AKI mouse models. Next, we modified the membrane of human placenta MSC‐derived EVs with a P‐selectin binding peptide (PBP, CDAEWVDVS) by conjugating PBP with a polyethylene glycol‐conjugated phospholipid derivative (DMPE‐PEG) to fabricate PBP‐engineered EVs (PBP‐EVs) with P‐selectin targeting ability. Furthermore, we explored the diagnostic potential and nephroprotective functions of PBP‐EVs after verifying their targeting ability with molecular imaging approaches. Overall, our study developed a proof‐of‐concept EV‐based theranostic strategy by targeting P‐selectin to specifically bind injured ECs in the kidney to monitor and protect against ischemic AKI in the early stage.

## 2 Results

### 2.1 P‐Selectin Expression in Ischemic AKI

**Figure 1:**
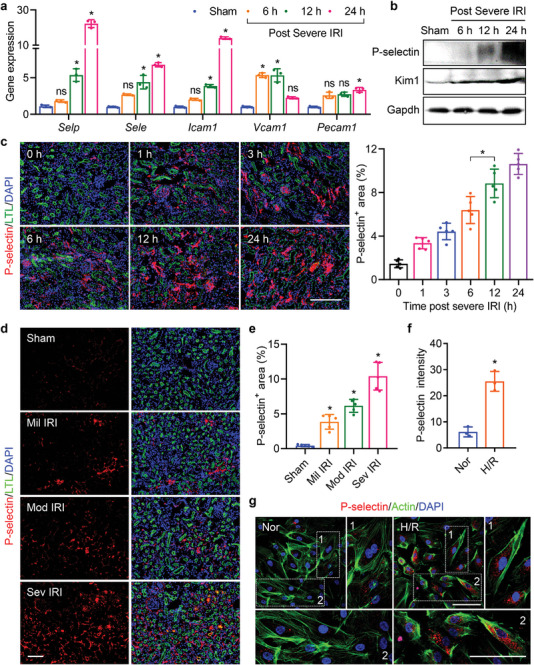
Expression of P‐selectin in the kidneys post AKI. a) Gene expression of *Selp*, *Sele*, *Icam1*, *Vcam1*, and *Pecam1* in renal tissues at 6, 12, and 24 h postsevere IRI. The renal tissues subjected to the sham operation served as the control, *n* = 3. ns, not significant versus sham, ^∗^ *P* < 0.05 versus sham. b) Western blot analysis of P‐selectin and Kim1 in renal tissues at 6, 12, and 24 h postsevere IRI (normalized to Gapdh). c) P‐selectin (red) expression in kidneys (FITC‐labeled Lotus tetragonolobus lectin (LTL), green, proximal tubules) at 0, 1, 3, 6, 12, and 24 h postsevere IRI was quantified by immunofluorescence, *n* = 5. ^∗^ *P* < 0.05. d) Immunofluorescence of P‐selectin (red) in kidneys (FITC‐labeled LTL, green, proximal tubules) at 12 h postmild, moderate, and severe IRI. e) Quantification of the P‐selectin‐positive area in renal sections, *n* = 5. *P < 0.05 versus sham. f) Average fluorescence intensity of P‐selectin in HUVECs post H/R, *n* = 3. *P < 0.05. g) P‐selectin (red) expression in HUVECs (Alexa Fluor 488‐labeled phalloidin, green, actin) post H/R was revealed by immunofluorescence. All data are expressed as the mean ± s.d. For a), c), and e), statistical analysis was performed using one‐way ANOVA with Tukey's multiple comparison tests. For f), statistical analysis was performed using a two‐tailed unpaired Student's *t*‐test. The nuclei were counterstained with DAPI (blue). Scale bars, 100 µm.

To screen feasible molecular targets for EC‐specific binding, we first examined the gene expression of several adhesion molecules (including *Selp*, *Sele*, *Icam1*, *Vcam1*, and *Pecam1*) in kidneys that are mainly responsible for leukocyte capture in the early stages of AKI.^[^ ^4d^ ^]^ We found that the expression levels of *Selp*, *Sele*, and *Icam1* were markedly increased in renal tissues after severe IRI, and the expression of *Selp* was the highest (**Figure** **1**a). Meanwhile, Western blot results confirmed that P‐selectin expression gradually increased with prolonged reperfusion time post severe IRI (Figure 1b). Immunofluorescence of P‐selectin in the injured kidneys further showed that P‐selectin was markedly increased at 12 h after reperfusion and widely distributed in glomeruli and peritubular vessels (Figure 1c). Subsequently, mild IRI, moderate IRI, and severe IRI models were established to examine the prevalence of P‐selectin in kidneys with different severities of injury (Figure S1a–c, Supporting Information). We found that P‐selectin expression was increased in all three IRI models and directly proportional to the severity of renal injury (Figure 1d,e). In addition, immunofluorescence of P‐selectin in human umbilical vein endothelial cells (HUVECs) after hypoxia/reoxygenation (H/R) injury verified in vitro that the expression of P‐selectin was significantly increased in injured ECs after hypoxia for 6 h and reoxygenation for 12 h (Figure 1f; and Figure S2, Supporting Information). These results suggested that the highly expressed P‐selectin in injured ECs is a feasible target for injured EC‐targeted renal therapy in the early stage of AKI.

### 2.2 Fabrication of the P‐Selectin‐Targeted EVs

**Figure 2:**
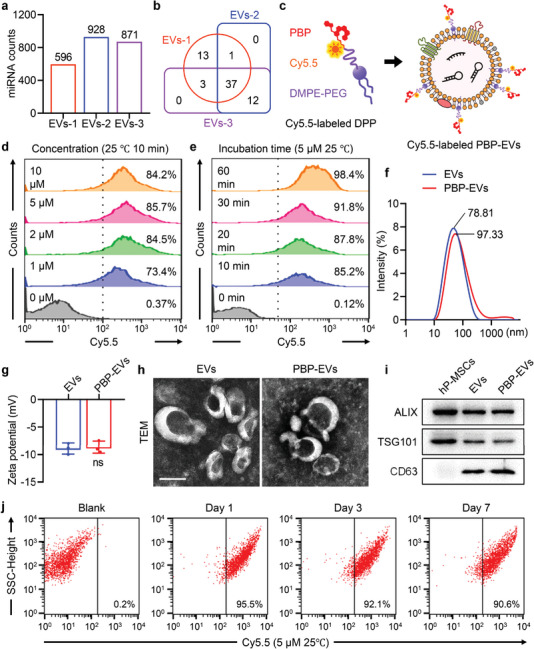
Fabrication and characterization of PBP‐EVs. a) Landscape atlas of miRNAs within EVs derived from three different donors. b) Venn diagram of abundantly expressed miRNAs identified in EVs derived from three donors. c) Schematic illustration of PBP‐EV fabrication through anchoring PBP on the EV membrane by DMPE‐PEG. Cy5.5 was conjugated on PBP side chains to visualize the surface modification of DMPE‐PEG‐PBP (DPP). d) Flow cytometry to assess the modification of Cy5.5‐labeled DPP with different molar concentrations on EVs. e) Flow cytometry to assess the efficiency of Cy5.5‐labeled DPP modification on EVs after incubation for different times. f) Size distributions of EVs and PBP‐EVs. g) Zeta potentials of EVs and PBP‐EVs, *n* = 3. ns, not significant. Statistical analysis was performed using a two‐tailed unpaired Student's *t*‐test. h) Transmission electron microscopy (TEM) images of EVs and PBP‐EVs. Scale bar, 100 nm. i) Western blot analysis confirmed the three categories of EV markers (ALIX, TSG101, and CD63) in EVs and PBP‐EVs. The hP‐MSC lysate served as a control. j) Flow cytometry to evaluate the stability of PBP‐EVs preserved at 4 °C for 1, 3, and 7 days.

To achieve injured EC‐targeted renal therapy, we designed P‐selectin‐targeted EVs by using hP‐MSC‐derived EVs, which have a superior nephroprotective function. First, we validated the beneficial microRNA (miRNA) components of the EVs that we isolated from the conditioned media of hP‐MSCs. According to the results of miRNA sequencing, we found that 1056 miRNAs, including 547 commonly expressed miRNAs, were detected in the 3 EV samples isolated from 3 independent donors (**Figure** **2**a; and Figure S3a, Supporting Information). All miRNAs were ranked according to read counts, and read counts greater than 1000 from each EV sample were considered abundantly expressed miRNAs. The intersection of abundantly expressed miRNAs from each EV sample yielded 37 common top miRNAs (Figure 2b; and Figure S3b,c, Supporting Information). The top 37 miRNAs accounted for 86.7% of all miRNAs EV contented, which were deemed to have major biological effects for further analysis compared to other miRNAs (Figure S3d, Supporting Information). The top 37 miRNAs were predicted to target 1685 genes with high stringency that overlapped in three miRNA databases (miRDB, TargetScan, and miRecords). The KEGG pathway analysis of predicted target genes indicated that the top 37 miRNAs substantially regulated signaling pathways mainly related to cell death and proliferation, angiogenesis, and fibrosis (Figures S4 and S5, Supporting Information). The gene ontology (GO) enrichment analysis for predicted target genes also suggested that these target genes participated in a number of biological processes related to renal regeneration, including positive regulation of cell proliferation, angiogenesis, regulation of the cell cycle, response to hypoxia, and several fibrosis‐related processes (Figure S6, Supporting Information). The top 37 miRNAs were classified into 6 categories according to their therapeutic functions (proliferation, antiapoptosis, proangiogenic, antifibrotic, antioxidative stress, and anti‐inflammatory) in renal regeneration based on previous reports (Figure S7, Supporting Information). These results validated that hP‐MSC‐derived EVs contained therapeutic miRNA components to achieve a potential nephroprotective effect.

Subsequently, we modified the P‐selectin‐binding peptide (PBP) on the surface of EVs by using a hydrophobic insertion strategy to fabricate PBP‐engineered EVs (PBP‐EVs) with the ability to identify and bind P‐selectin on injured ECs (Figure 2c). PBP (CDAEWVDVS) was covalently bound to DMPE‐PEG5000‐maleimide (DMPE‐PEG‐MAL) to synthesize DMPE‐PEG‐PBP (DPP), which could be inserted into the membrane of EVs (Figure S8a, Supporting Information). The successful synthesis of DPP was verified by the presence of the PBP indole ring (7.0–7.6 ppm region) in the spectra of DPP in 1H NMR analysis (Figure S8b, Supporting Information). Cyanine 5.5 (Cy5.5) was coupled to the PBP for visualization. Flow cytometry (FCM) could distinguish EVs from background noise by no overlap between the populations of EVs and DPP or PBS (Figure S9a, Supporting Information). The FCM of EVs incubated with different concentrations of Cy5.5‐labeled DPP for 10 min at 25 °C indicated that modification efficiency was positively associated with the concentration of DPP and plateaued at a concentration of 5 µm (Figure 2d). Meanwhile, the FCM of EVs incubated with 5 µm Cy5.5‐labeled DPP for different times at different temperatures revealed that modification efficiency increased with prolonged incubation time and increased incubation temperature (Figure 2e; and Figure S9b, Supporting Information). Considering the efficiency and operability of the modification, we incubated EVs with 5 µm DPP at room temperature (25 °C) for 30 min to fabricate PBP‐EVs, and their positive rate was maintained above 90%. Subsequently, the characterization of PBP‐EVs was performed. Compared with EVs, the zeta potential of PBP‐EVs was unchanged, but the particle size was slightly increased (Figure 2f,g). The morphology of PBP‐EVs did not change and was still membrane‐bound round‐shaped vesicles (Figure 2h). Western blot bands showed that three protein markers of EVs (Alix, TSG101, and CD63) were still expressed in PBP‐EVs (Figure 2i). There were no significant differences in the total RNA and miR‐21‐5p amounts in EVs and PBP‐EVs, demonstrating that DPP modification did not influence the RNA cargos in EVs (Figure S9c, Supporting Information). Finally, the stability of PBP‐EVs preserved at 4 °C for 1, 3, and 7 days was tested by FCM. The 90% positive rate of PBP‐EVs stored for 7 days suggested that DPP modification remained stable for 1 week when kept undisturbed at 4 °C (Figure 2j). Moreover, both the size distribution and zeta potential of PBP‐EVs did not change after 7 days of storage at 4 °C (Figure S9d, Supporting Information). In summary, we fabricated P‐selectin‐targeted PBP‐EVs by inserting PBP into the membrane of hP‐MSC‐derived EVs without losing the intrinsic properties of natural EVs.

### 2.3 PBP‐EVs Showed Preferential Renal Targeting Ability by Binding Injured ECs

**Figure 3:**
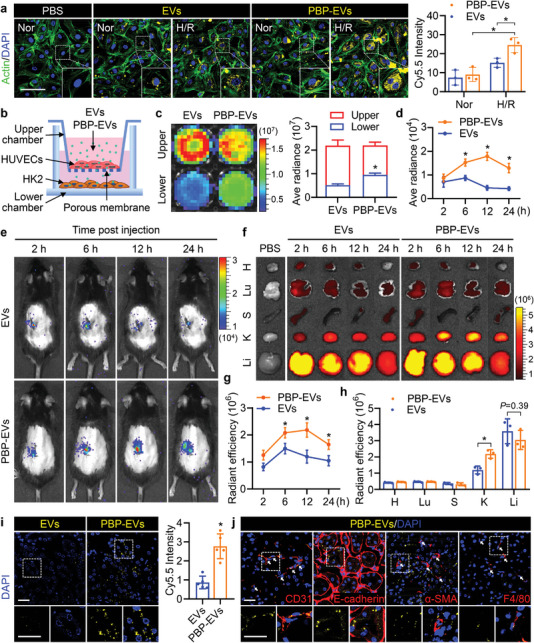
Endothelial cell targeting capacity of PBP‐EVs. a) H/R‐injured HUVECs (Alexa Fluor 488‐labeled phalloidin, green, actin) demonstrated enhanced internalization of PBP‐EVs (Cy5.5, yellow) in vitro. Scale bars, 100 µm, *n* = 3. b) Diagram of a modified Transwell assay to detect penetration of EVs or PBP‐EVs into an H/R‐injured endothelial monolayer barrier (endothelial cells, HUVECs) and infiltration into renal parenchyma cells (tubular epithelial cells, HK2). c) Gluc signals of EVs and PBP‐EVs in the upper and lower chambers of the modified Transwell system were evaluated by BLI, *n* = 3. d) Quantitative analysis of EVs and PBP‐EVs accumulated in the injured kidney by Gluc imaging. The average radiance of the Gluc signals was expressed as photons/s/cm^2^/steradian, *n* = 3. e) The renal targeting ability of PBP‐EVs was traced in real time in vivo by Gluc imaging. f) Organ distributions of EVs and PBP‐EVs in severe IRI mice were detected by Cy5.5 radiant efficiency after a single intravenous injection of 100 µg EVs or PBP‐EVs. H: heart, Lu: lung, S: spleen, K: kidney, Li: liver. g) Quantitative analysis of EVs and PBP‐EVs accumulated in the injured kidney by Cy5.5 signals at 2, 6, 12, and 24 h after injection. The radiant efficiency of Cy5.5 was expressed as [photons/s/cm^2^/steradian]/[µW cm^−2^], *n* = 3. h) Quantitative analysis of Cy5.5 radiances in the indicated organs of severe IRI mice at 12 h after injection. The radiant efficiency of Cy5.5 was expressed as [photons/s/cm^2^/steradian]/[µW cm^−2^], *n* = 3. H: heart, Lu: lung, S: spleen, K: kidney, Li: liver. i) Representative images and quantitative analysis of EVs and PBP‐EVs (Cy5.5, yellow) accumulated in injured renal tissues 12 h postinjection. Scale bar, 25 µm, *n* = 3. j) Representative images showing the accumulation of PBP‐EVs (Cy5.5, yellow) in endothelial cells (CD31^+^, red), tubular epithelial cells (E‐cadherin^+^, red), fibroblasts (*α*‐SMA^+^, red), and macrophages (F4/80^+^, red) in injured renal tissues 12 h postinjection. The bottom was a higher magnification of the boxed region. Scale bar, 25 µm. All data are expressed as the mean ± s.d. For a), c), d), and g), statistical analysis was performed using two‐way ANOVA with Tukey's multiple comparison tests. For h) and i), statistical analysis was performed using two‐tailed unpaired Student's *t*‐tests. *P < 0.05. The nuclei were counterstained with DAPI (blue).

To visualize EVs and PBP‐EVs in vitro and vivo, we fabricated Cy5.5‐ and Gaussia luciferase (Cy5.5/Gluc)‐labeled EVs and PBP‐EVs (Figure S10a–c, Supporting Information). The targeting selectivity of PBP‐EVs to P‐selectin was evaluated in H/R‐injured HUVECs that highly expressed P‐selectin (Figure S11a, Supporting Information). After incubation with Cy5.5/Gluc‐labeled EVs and PBP‐EVs for 2 h, Gluc imaging showed that H/R‐injured HUVECs internalized significantly more PBP‐EVs than EVs (Figure S11b, Supporting Information). Fluorescent images of Cy5.5 in HUVECs revealed the same results, suggesting that more PBP‐EVs could be internalized by H/R‐injured HUVECs by binding to P‐selectin (**Figure** **3**a). Subsequently, the ability of PBP‐EVs to penetrate through the injured ECs into tubular epithelial cells (TECs) was evaluated using a modified Transwell system (Figure 3b). HUVECs (upper chambers) and HK2 cells (lower chambers) cocultured in Transwell systems were injured by H/R, and then Cy5.5/Gluc‐labeled EVs or PBP‐EVs were added to the upper chambers (Figure S11c, Supporting Information). After incubation for 6 h, the Gluc radiance of PBP‐EVs collected from the lower chambers was two times higher than that of EVs (Figure 3c). Fluorescent images of HK2 also revealed that the penetration of PBP‐EVs across injured ECs was increased compared to EVs, which resulted in enhanced uptake by HK2 (Figure S11d, Supporting Information).

Encouraged by the above findings, we further explored the kidney targeting ability of PBP‐EVs in severe IRI mice (Figure S12a, Supporting Information). After reperfusion for 12 h, when the level of P‐selectin in injured kidneys was already elevated, 100 µg of Gluc‐labeled EVs, and PBP‐EVs were intravenously injected. Bioluminescence imaging showed that the Gluc radiances of the injured kidney region in the PBP‐EV group were remarkably higher than those in the EV group at 6, 12, and 24 h after injection, which indicated that PBP‐EVs could specifically target the injured kidneys (Figure 3d,e). In addition, the organ distributions of Cy5.5‐labeled EVs and PBP‐EVs in severe IRI mice were evaluated by fluorescence imaging at 2, 6, 12, and 24 h after intravenous injection. An obvious increase in Cy5.5 radiant efficiency was detected in the injured kidneys of PBP‐EV‐injected mice compared to EVs, suggesting that PBP can specifically redirect the systemic distribution of PBP‐EVs to the injured kidneys (Figure 3f–h). Subsequently, the preferential nephrontropism of PBP‐EVs was verified in renal sections that had accumulated abundant PBP‐EVs (Figure 3i; and Figure S12b, Supporting Information). The cell distribution of PBP‐EVs in the injured kidney was further explored by immunofluorescence at 12 h postinjection. We found robust deposition of PBP‐EVs in ECs (CD31^+^) and TECs (E‐cadherin^+^) rather than in fibroblasts (*α*‐SMA^+^) and macrophages (F4/80^+^) (Figure 3j). Together, these results demonstrated that PBP‐EVs exhibited a preferential ability to target injured kidneys by binding to P‐selectin on the injured ECs and accumulated primarily in ECs and TECs.

### 2.4 PBP‐EVs Could be Used for Monitoring the Severity of AKI in the Early Stage

**Figure 4:**
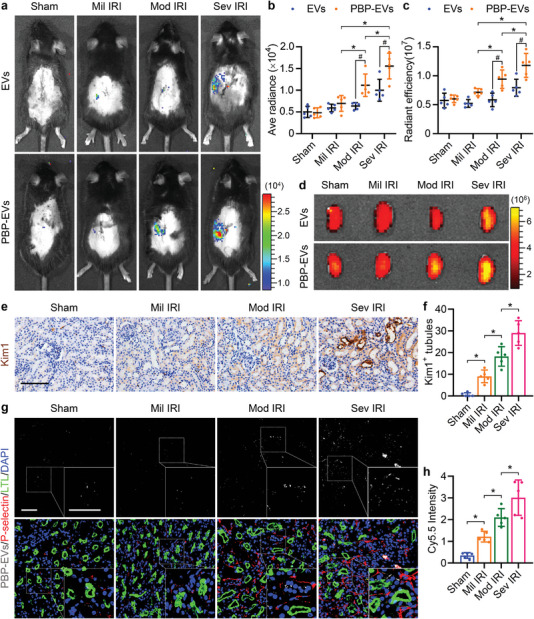
Potential of PBP‐EVs to monitor the severity of AKI. a) Gluc imaging of PBP‐EVs 12 h postinjection revealed different degrees of renal injury induced by mild IRI, moderate IRI, and severe IRI. b) Quantitative analysis of Gluc radiances from EVs and PBP‐EVs in the kidneys with varying degrees of injury. The average radiance of Gluc was expressed as photons/s/cm^2^/steradian, *n* = 5. c) Quantitative analysis of Cy5.5 radiances from EVs and PBP‐EVs in the kidneys with varying degrees of injury 12 h after injection. The radiant efficiency of Cy5.5 was expressed as [photons/s/cm^2^/steradian]/[µW cm^−2^], *n* = 5. d) Accumulation of EVs and PBP‐EVs in the kidneys after mild IRI, moderate IRI, or severe IRI was detected by Cy5.5 radiant efficiency at 12 h after a single intravenous injection of 100 µg EVs or PBP‐EVs. e) Immunohistochemistry of Kim1 in renal tissues 12 h postinjection after mild IRI, moderate IRI, or severe IRI. Scale bars, 100 µm. f) Quantitative analysis of Kim1^+^ renal tubules on immunohistochemical images, *n* = 5. g) PBP‐EV (Cy5.5, gray) accumulation and P‐selectin expression (red) in renal tissues 12 h postinjection (FITC‐labeled LTL, green, proximal tubules) after mild IRI, moderate IRI, or severe IRI. Scale bars, 50 µm. h) PBP‐EV accumulation in renal tissues after mild IRI, moderate IRI, or severe IRI was measured by Cy5.5 radiant efficiency, *n* = 5. All data are expressed as the mean ± s.d. For b) and c), statistical analysis was performed using two‐way ANOVA with Tukey's multiple comparison tests. For f) and h), statistical analysis was performed using one‐way ANOVA with Tukey's multiple comparison tests. *P < 0.05. ^#^ *P* < 0.05 versus EVs. The nuclei were counterstained with DAPI (blue).

Subsequently, we explored whether PBP‐EVs had the potential to monitor the severity of AKI in mild IRI, moderate IRI, and severe IRI models by intravenous injection of 100 µg Cy5.5/Gluc‐labeled EVs or PBP‐EVs per mouse 12 h postreperfusion (Figure S13a, Supporting Information). Bioluminescence imaging of the above mice 12 h after injection showed that the Gluc radiance of PBP‐EVs in the injured kidney regions was enhanced gradually as the severity of IRI increased (**Figure** **4**a,b). To assess the precision of the Gluc imaging results, these injured kidneys were exteriorized for Cy5.5 fluorescence imaging. Consistent with the Gluc imaging results, there was an obvious correlation between the Cy5.5 radiance of PBP‐EVs and the severity of renal IRI, while increased Cy5.5 radiance from EVs was observed only in kidneys with severe IRI (Figure 4c,d). The immunofluorescence of kidney injury molecule 1 (Kim1) of the kidneys from the PBP‐EVs group revealed that the number of Kim1^+^ tubules in the injured kidneys was also in step with the Cy5.5 radiance of PBP‐EVs (Figure 4e,f). To accurately analyze the relationship between the Gluc or Cy5.5 radiance and the severity of kidney injury, we performed linear regression analysis on the Gluc or Cy5.5 radiance and the number of Kim1^+^ tubules. According to the linear analysis results, we found that both Gluc and Cy5.5 radiance from PBP‐EVs showed a relatively higher R‐Squared (>0.77) with the number of Kim1^+^ tubules than radiance from EVs (0.32–0.56) (Figure S13b,c, Supporting Information). The higher accuracy of the goodness of fit measures from the PBP‐EV model confirmed that PBP‐EVs could be a more accurate indicator for the severity of renal injury than EVs. P‐selectin immunofluorescence of these renal cryosections further demonstrated that the Cy5.5 radiance of PBP‐EVs, which increased with the severity of renal IRI, was due to the increase in P‐selectin in the injured ECs, which were specifically targeted by PBP‐EVs (Figure 4g,h). In conclusion, the radiance from PBP‐EVs could indicate the severity of renal injury within 24 h by quantifying the expression of P‐selectin in injured kidneys, which is a promising candidate for the early diagnosis of ischemic AKI.

### 2.5 PBP‐EVs Reversed Renal Microvascular Dysfunction

**Figure 5:**
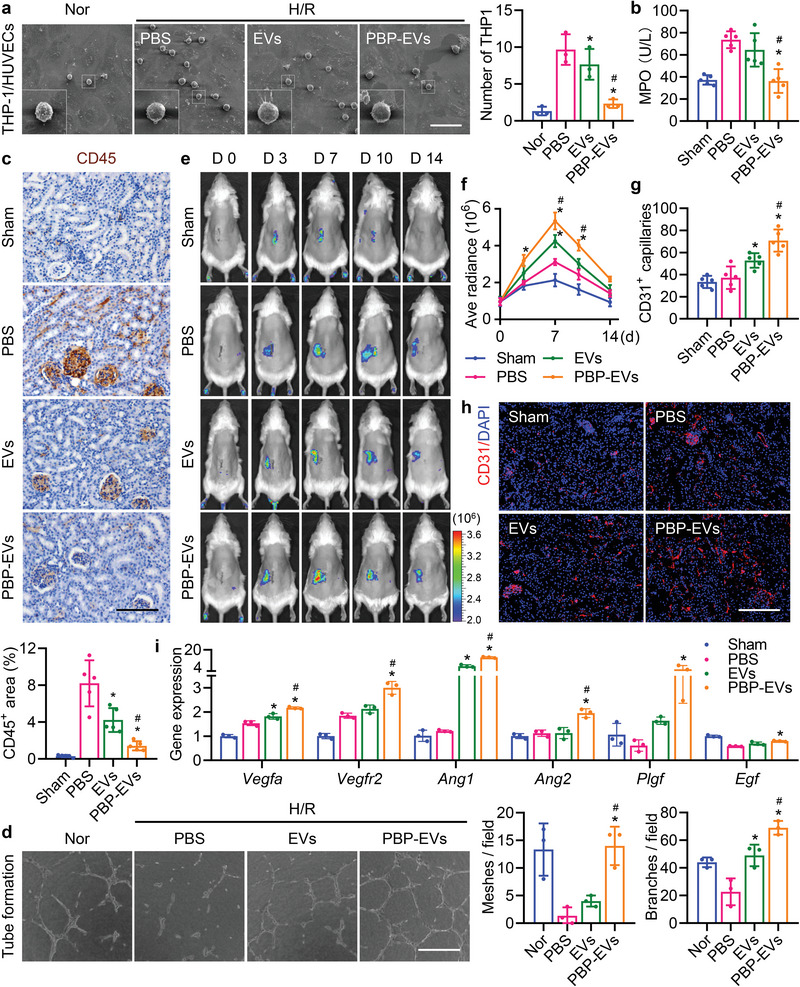
PBP‐EVs regulate endothelial cell functions. a) Scanning electron microscopy (SEM) images of human monocyte THP1 binding to H/R‐injured HUVECs with or without PBP‐EV blockade. Scale bar, 50 µm, *n* = 3. b) Myeloperoxidase (MPO) activity in renal tissue homogenate from different groups at day 3 postsevere IRI, *n* = 5. c) Immunohistochemistry and quantification of CD45 (brown) in renal tissues from different groups on day 3 postsevere IRI. Scale bar, 100 µm, *n* = 5. d) Representative images and quantification (numbers of meshes and branches per field) of the networks formed by HUVECs treated with EVs or PBP‐EVs for 6 h. Scale bar, 200 µm, *n* = 3. e) Vegfr2 expression in injured kidneys was monitored in real time by Fluc imaging in Vegfr2‐Fluc KI mice. f) Angiogenic tendencies of injured kidneys were quantitatively analyzed by Fluc radiance. The average radiance of Fluc was expressed as photons/s/cm^2^/steradian, *n* = 3. g) Quantitative analysis of CD31^+^ capillaries of injured renal tissues in immunofluorescence images, *n* = 5. h) The capillaries of the injured renal tissue were counted by immunofluorescence with CD31 (red) on day 7 postsevere IRI. The nuclei were counterstained with DAPI (blue). Scale bar, 200 µm. i) Angiogenesis‐related gene expression (*Vegfa*, *Vegfr2*, *Ang1*, *Ang2*, *Plgf*, and *Egf*) in renal tissues was assessed by real‐time qPCR on day 7 postsevere IRI. The renal tissues subjected to the sham operation served as the control, *n* = 3. All data are expressed as the mean ± s.d. For a–d), g), and i), statistical analysis was performed using one‐way ANOVA with Tukey's multiple comparison tests. For f), statistical analysis was performed using two‐way ANOVA with Tukey's multiple comparison tests. *P < 0.05 versus PBS, ^#^ *P* < 0.05 versus EVs.

After confirming the diagnostic ability of PBP‐EVs, we focused on their therapeutic functions to protect against AKI. As the first cell type to signal to leukocytes, injured ECs trigger the immune response, which means that dynamic interactions between injured ECs and leukocytes play a crucial role in IRI‐induced inflammation. P‐selectin is a critical molecular basis of leukocyte tethering and rolling on the injured vascular wall‐mediated leukocyte adhesion and infiltration in injured kidneys; after PBP‐EVs are intravenously injected, P‐selectin may be competitively bound by injected PBP‐EVs. Therefore, we examined the blocking function of PBP‐EVs on the adhesion of monocytes to injured ECs in vitro. Scanning electron microscopy (SEM) images revealed that a large number of human THP‐1 monocytes adhered to the surface of H/R‐injured HUVECs through numerous filopodia, while PBP‐EVs significantly reduced the number of adherent THP‐1 cells on the surface of HUVECs (**Figure** **5**a). Furthermore, myeloperoxidase activity and CD45^+^ cells in renal tissues confirmed in vivo that PBP‐EVs could effectively reduce leukocyte infiltration in injured kidneys at day 3 after severe IRI, echoing the in vitro SEM results of reduced leukocyte adhesion on the injured EC surface (Figure 5b,c).

Meanwhile, EC injury could also lead to failure of reparative angiogenesis and rarefaction of the vasculature, which is the pivotal cause of the hypoxic niche present in the kidneys after IRI. Accordingly, we detected the beneficial effects of PBP‐EVs in vitro by tube formation and wound healing assays (Figure S14a,b, Supporting Information). It was impressive that the angiogenic capacities, including tube formation and migration of HUVECs destroyed by H/R injury, could be rescued and even improved after PBP‐EV administration (Figure 5d; and Figure S14c,d, Supporting Information). In light of these findings, we further investigated reparative angiogenesis in injured kidneys after PBP‐EV treatment using angiogenesis reporter transgenic mice, Vegfr2‐Fluc‐KI mice, which express firefly luciferase (Fluc) in a manner consistent with the endogenous expression of Vegfr2. Bioluminescence imaging revealed that Fluc radiance increased and peaked at day 7 postsevere IRI in all groups, and the enhanced proangiogenic ability of PBP‐EVs was manifested by the strongest Fluc radiance from PBP‐EV‐treated mice (Figure 5e,f). Moreover, the increased CD31^+^ capillaries on day 7 postsevere IRI verified the superior neovascularization in kidneys post PBP‐EV treatment (Figure 5g,h). Expression analysis for angiogenesis‐related genes, including *Vegfa*, *Vegfr2*, *Ang1*, *Ang2*, *Plgf*, and *Egf*, reflected the changes seen in renal sections, which demonstrated that PBP‐EV treatment strongly promoted reparative angiogenesis (Figure 5i). Collectively, PBP‐EVs that specifically bind and enter endothelial cells could decrease inflammatory cell infiltration while increasing reparative angiogenesis in the injured kidneys after severe IRI.

### 2.6 PBP‐EVs Repaired the Renal Parenchymal Lesion

**Figure 6:**
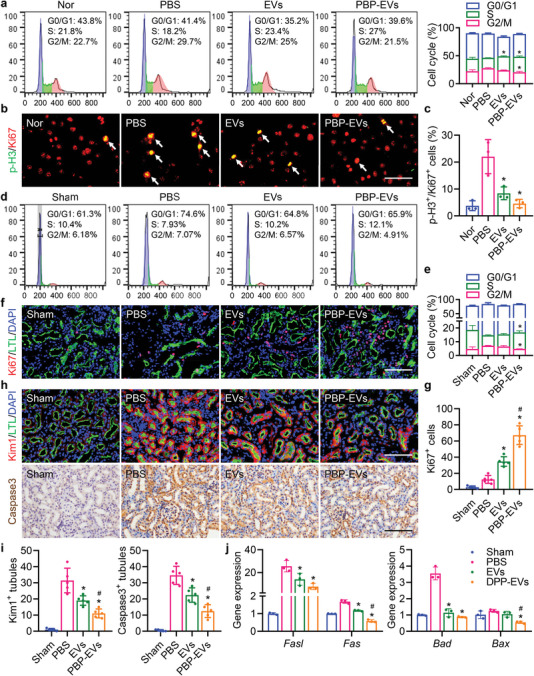
PBP‐EVs ameliorate maladaptive repair of tubular epithelial cells. a) The cell cycle of H/R‐injured HK2 cells was assessed by PI staining after administration of EVs or PBP‐EVs for 24 h. Normal HK2 cells served as a control, *n* = 3. b) Immunofluorescence of Ki67 (red) and p‐H3 (green) in H/R‐injured HK2 cells treated with EVs or PBP‐EVs for 24 h. Scale bar, 50 µm. c) Percentage of proliferating HK2 (Ki67^+^) cells that were in the G2/M phase (p‐H3^+^) of the cell cycle after H/R injury with administration of EVs or PBP‐EVs, *n* = 3. d) Cell cycles of TECs isolated from injured kidneys on day 3 postsevere IRI were analyzed by PI staining. Cells isolated from kidneys with sham operation served as a control. e) Percentage of TECs isolated from kidneys according to the cell cycle distribution, *n* = 3. f) Immunofluorescence of Ki67 (red) in renal tissues (FITC‐labeled LTL, green, proximal tubules) on day 3 postsevere IRI. Scale bar, 100 µm. g) Quantification of Ki67^+^ cells in renal immunofluorescence images, *n* = 5. h) Immunofluorescence of Kim1 (red, top row) and caspase3 (brown, bottom row) in renal tissues at day 3 postsevere IRI. Scale bar, 100 µm. i) Quantitative analysis of Kim1^+^ renal tubules and caspase3^+^ renal tubules in immunofluorescence images. *n* = 5. j) Expression of apoptosis‐related genes (*Fasl*, *Fas*, *Bad*, and *Bax*) in renal tissues was detected by real‐time qPCR on day 3 postsevere IRI. The renal tissues subjected to the sham operation served as the control, *n* = 3. All data are expressed as the mean ± s.d. Statistical analysis was performed using one‐way ANOVA with Tukey's multiple comparison tests. *P < 0.05 versus PBS, ^#^ *P* < 0.05 versus EVs. The nuclei were counterstained with DAPI (blue).

In addition, proximal TECs are highly susceptible to IRI and are prone to ischemic injury and consequent apoptosis. Dysfunction and apoptosis of these TECs are primarily responsible for the pathophysiological consequences and clinical symptoms of IRI‐induced AKI, although EC injury is also prominent and partly mediates inflammation and long‐term consequences. Our results also confirmed that a large number of PBP‐EVs penetrated through the injured endothelium and were internalized by TECs. In this context, we further evaluated the impact of PBP‐EVs on the cell cycle of TECs injured by I/R in vitro, which is a critical process during tubular repair (Figure S15a, Supporting Information). The results revealed that the cell cycle of HK2 was arrested in the G2/M phase after H/R injury (27.8%), while the percentages of HK2 in the G2/M phases decreased after EV or PBP‐EV treatments (20.4%). Furthermore, HK2 cells showed a stronger proliferative capacity after PBP‐EV treatment, manifested by the significantly increased percentages of HK2 cells in the S phase (27.73%) and rapidly passing through the G2/M checkpoint into the next cell cycle (**Figure** **6**a). This finding was also evidenced by a decrease in the percentage of HK2 with phosphorylation of histone H3 at Ser10 (p‐H3; G2/M phase marker) in Ki67^+^ HK2 (G1, S, G2, and M phase) (Figure 6b,c). Then, we detected the cell cycle of TECs (E‐cadherin^+^) isolated from the injured kidneys on day 3 postsevere IRI (Figure S15b,c, Supporting Information). Consistent with the phenomenon observed in vitro, PBP‐EVs rescued the G2/M arrest caused by severe IRI and distinctly promoted the proliferation of HK2 cells (Figure 6d,e). The number of Ki67^+^ TECs in renal tissues on day 3 postsevere IRI reconfirmed that PBP‐EVs treatment discernibly promoted TECs proliferation compared to EVs, reflecting an improved repair response (Figure 6f,g). Furthermore, immunofluorescence of Kim1 and caspase3 in injured kidneys indicated that the G2/M arrest of TECs led to maladaptive repair, resulting in the failure of tubule regeneration and accumulation of large numbers of injured and apoptotic proximal tubules in the kidneys, while PBP‐EVs effectively decreased the number of Kim1^+^ and Caspase3^+^ tubules by promoting TEC proliferation (Figure 6h,i). Assessment of apoptosis‐related genes (*Fasl*, *Fas*, *Bad*, and *Bax*) corroborated our findings that PBP‐EVs significantly attenuated severe IRI‐induced apoptosis, echoing the staining results (Figure 6j). Overall, PBP‐EVs promoted regeneration and repaired proximal tubules by advancing the cell cycle progression of TECs.

### 2.7 PBP‐EVs Accelerated Renal Recovery and Prevented the Progression of AKI to CKD

**Figure 7:**
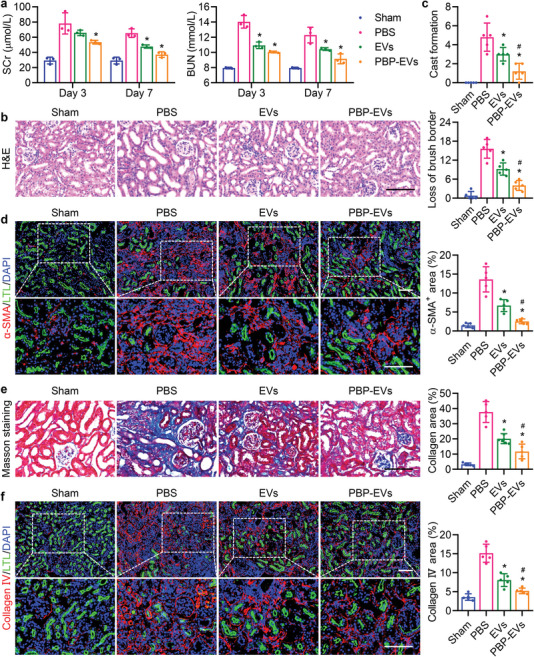
PBP‐EVs facilitated renal recovery and alleviated fibrosis. a) Serum creatinine (SCr) and blood urea nitrogen (BUN) were evaluated on days 3 and 7 postsevere IRI, *n* = 3. b) Hematoxylin and eosin (H&E) staining of renal tissues 3 days postsevere IRI. c) Quantification of cast formation and loss of brush border in H&E staining image, *n* = 5. d) *α*‐SMA (red) expression in renal tissues (FITC‐labeled LTL, green, proximal tubules) at day 28 postsevere IRI was quantified by immunofluorescence, *n* = 5. e) Collagen fibrils in renal tissues 28 days after severe IRI were visualized and quantified by Masson's trichrome staining, *n* = 5. f) Collagen IV (red) expression in renal tissues (FITC‐labeled LTL, green, proximal tubules) at day 28 postsevere IRI was quantified by immunofluorescence, *n* = 5. All data are expressed as the mean ± s.d. For a), statistical analysis was performed using two‐way ANOVA with Tukey's multiple comparison tests. For c– e), and f), statistical analysis was performed using one‐way ANOVA with Tukey's multiple comparison tests. *P < 0.05 versus PBS, ^#^ *P* < 0.05 versus EVs. The nuclei were counterstained with DAPI (blue). Scale bar, 100 µm.

To further validate the nephroprotective effects of PBP‐EVs, we examined the function and structure of the injured kidneys after severe IRI. Renal function analysis at days 3 and 7 post IRI, including blood urea nitrogen (BUN) and serum creatinine (SCr), indicated that IRI led to a deterioration of renal function characterized by elevated concentrations of BUN and SCr. The lowest concentrations of BUN and SCr after treatment showed that PBP‐EVs exhibited better beneficial effects than EVs and remarkably alleviated renal failure (**Figure** **7**a). Hematoxylin and eosin (H&E) staining of renal tissues at day 3 post IRI to evaluate the histological structure. After severe IRI, massive cast formation and necrotic tubules accompanied by brush border loss that appeared in injured kidneys were obviously reduced after PBP‐EV treatment (Figure 7b,c; and Figure S16a, Supporting Information). Quantification of a panel of kidney injury marker genes supported our histopathological observations that PBP‐EVs significantly attenuated kidney damage markers (*Kim1*, *Ngal*, *Nphs1*, and *Nphs2*), resulting in levels comparable to those in the sham groups (Figure S16b, Supporting Information).

In addition, the expression of profibrogenic cytokines (*Ctgf*, *Pdgf*, and *Tgfb1*), which are responsible for the activation of fibroblasts to myofibroblasts, was reduced in the injured kidneys after 3 days of treatment with PBP‐EVs (Figure S17a, Supporting Information). This finding indicated that PBP‐EVs not only accelerated renal regeneration in the early stage of AKI but also inhibited myofibroblast activation during the extension phase of AKI, allowing us to speculate that the profibrotic microenvironment in the injured kidneys had been resolved by PBP‐EVs through decreasing infiltration of inflammatory cells, increasing reparative angiogenesis, and ameliorating renal parenchymal lesions. As a result, the expression of fibrosis‐related genes (*Acta1*, *Col1a1*, and *Col4a1*) in the kidneys at days 3, 7, and 28 postsevere IRI was accordingly inhibited by treatment with PBP‐EVs (Figure S17b, Supporting Information). Immunofluorescence of *α*‐SMA in renal tissues on day 28 postsevere IRI revealed that *α*‐SMA^+^ myofibroblasts were abundant in the renal interstitium post IRI and notably decreased after treatments with EVs and PBP‐EVs, validating our finding of an improved profibrotic microenvironment in injured kidneys (Figure 7d). Masson's trichrome staining showed a similar trend: collagen deposition in injured kidneys was markedly reduced after PBP‐EV treatment (Figure 7e; and Figure S17c, Supporting Information). Collagen IV, a marker of glomerular basement membrane thickening and dysfunction, was found to deposit around glomeruli and atrophic tubules after severe IRI. Quantification of the collagen IV area revealed that collagen IV deposition was reduced after PBP‐EV treatment, but thickened basement membranes were still observable (Figure 7f). Furthermore, the number of functional renal tubules (LTL^+^) was notably increased and was comparable to that of the sham group after PBP‐EV treatment. Collectively, these data demonstrated that PBP‐EVs accelerated renal recovery and alleviated fibrosis to protect against further progression of AKI to CKD.

Finally, the biosafety of PBP‐EVs was assessed by analysis of serum biochemical indices, including aspartate aminotransferase (AST) and alanine aminotransferase (ALT), and histological analysis of the main organs on day 28 postinjection (Figure S18a, Supporting Information). Serum biochemical analysis revealed that no significant differences were observed in AST and ALT after PBP‐EV injection (Figure S18b, Supporting Information). Histology of the heart, lung, liver, spleen, and intestine revealed no obvious alterations in all organs after injection of PBP‐EVs compared with the control, suggesting that there was no organ toxicity of PBP‐EVs (Figure S18c, Supporting Information). Taken together, these findings demonstrated that noninvasive intravenous injection of PBP‐EVs provided reliable biosafety for the early diagnosis and targeted therapy of AKI.

## 3 Discussion

**Figure 8:**
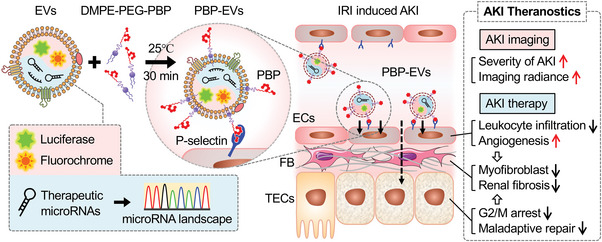
Graphical summary of the PBP‐EV‐based AKI theranostics. PBP‐EVs carry therapeutic miRNAs and imaging agents specifically targeting the injured kidney by binding to P‐selectin on injured endothelial cells, which can be used to monitor the severity of AKI while promoting renal recovery.

In this study, we first identified that P‐selectin expressed on injured ECs was a superior target for theranostics of ischemic AKI and designed P‐selectin‐targeted PBP‐EVs with binding selectivity to injured ECs for monitoring and treating IRI‐induced AKI. Molecular imaging revealed that PBP‐EVs exhibited preferable selectivity to injured kidneys and have the potential to monitor the severity of renal injury in real time at the early stage. Our results demonstrated that PBP‐EVs could inhibit inflammatory cell infiltration by competitively binding P‐selectin to injured ECs. In summary, PBP‐EVs significantly accelerated renal recovery and prevented the development of AKI to CKD through the reversion of the profibrogenic microenvironment in the kidneys (**Figure** **8**).

Endothelial injury and the switch of ECs from regulatory status to a prothrombotic and proinflammatory phenotype are common features of multiple renal diseases, not only IRI‐induced AKI.^[^ ^2^ ^]^ The initial alarm sounded immediately after EC injury triggers an immune response, which is then amplified by the recruitment of inflammatory cells, leading to ongoing hypoxia and profibrogenic microenvironments and further injury.^[^ ^9^ ^]^ Thus, identification of the initial alarm molecules is the key to developing an injured EC‐targeted theranostic strategy and minimizing the subsequent distal cascades. In our study, we selected renal IRI mice as a proof‐of‐principle model and identified P‐selectin as an adhesion molecule that is an initial alarm molecule and a candidate target for injured EC‐targeted renal therapy. Elevated P‐selectin expression, which was correlated with the extension of reperfusion time and severity of renal injury, was found in the glomeruli and peritubular vessels in the kidney after IRI. These results inspired us to design engineered EVs to selectively bind P‐selectin for EC‐targeted renal theranostic strategies.

To enable EVs to selectively identify and bind P‐selectin on injured ECs, we modified EV membranes with the consensus motif (E)WVDV of PBP. Compared to the antagonist or antibody of P‐selectin, PBP afforded unique advantages, including high‐throughput yields at a low cost benefiting from the contribution of the solid phase peptide synthesis technique and ease of conjugation or modification as required due to the relatively simplistic and well‐defined chemical structure of polypeptides.^[^ ^10^ ^]^ Additionally, it has been proven that PBP has a remarkable P‐selectin binding specificity with high affinity, which provides sufficient strength for anchoring PBP‐EVs on the surface of injured ECs under the wall shear of hemodynamic flow.^[^ ^10c^ ^]^ Therefore, we rationalized that PBP not only enhanced the selective specificity of PBP‐EVs but also provided spatiotemporal convenience for PBP‐EVs to access into and penetrate the injured endothelial layer in injured kidneys, which demonstrated that PBP is a superior targeting ligand for EC‐targeted renal theranostics.

Moreover, when designing engineered EVs for theranostics of AKI, we also need to consider a versatile and feasible approach for EV modification to facilitate their clinical transformation. Recently, several strategies have been developed to modify EVs with exogenous targeting ligands that can accurately deliver EVs to target tissues.^[^ ^11^ ^]^ Although proven to be successful, these strategies still have some limitations, including the potential risk of gene editing, unescapable restriction of enzyme catalysis sites, and possible cell toxicity of covalent coupling.^[^ ^12^ ^]^ Considering these reasons, we employed the polyethylene glycol‐conjugated phospholipid linker DMPE‐PEG to anchor PBP on the membranes of EVs through hydrophobic interactions. PBP‐EVs were fabricated using simple coincubation (at room temperature for 30 min) with a positive rate of up to 90%, showing consistent uniformity and reproducibility of this method. Furthermore, our results also demonstrated that the PBP‐EVs we fabricated using this method could be stored stably at 4 °C for at least 1 week. Overall, this simple, efficient, reproducible, and stable fabrication method of PBP‐EVs provided feasibility for the industrialized production of EV‐based theranostic systems and their large‐scale clinical applications.

Moreover, the imaging agents loaded in PBP‐EVs can monitor the severity of AKI and predict long‐term outcomes in real time by quantifying the accumulation of PBP‐EVs in the injured kidneys. This is of great significance since there are currently no adequate clinical diagnostic and quantification techniques to allow for early detection, injury quantification, and therapeutic evaluation for AKI. Accurately determining the severity of AKI in the early stage could directly prolong the exposure of the patient to receive therapeutic interventions that delay or attenuate adverse outcomes. However, the Gluc and Cy5.5 labeled on PBP‐EVs in this study still need to be replaced with clinical imaging agents with deeper penetration depths and higher resolutions, such as superparamagnetic iron oxide nanoparticles or radionuclides, in clinical applications.

Beyond the diagnostic potential, noninvasive injection of PBP‐EVs also showed impressive therapeutic functions in the IRI‐induced AKI model. Although our previous studies have found that stem cell‐derived EVs could produce beneficial effects in injured kidneys after invasive localized injection or repeat dosing, uncontrolled delivery can still increase the risk of biotoxicity.^[^ ^13^ ^]^ PBP‐EVs that showed excellent kidney‐targeted properties, smart accumulation patterns, and remarkable nephroprotective effects after a single dose injection could reduce this risk and exhibit striking superiority. As a proof of concept, therapeutic amounts of PBP‐EVs were achieved with a single dose of intravenous injection for rescuing renal function without any detectable toxicity, indicating that the PBP‐EVs were an efficient, smart, and safe EC targeted therapy for AKI.

Furthermore, one of the attractive features of using PBP‐EVs as targeted therapeutic carriers of AKI is that of the natural beneficial components contained in them. An increasing number of studies have confirmed that EVs derived from MSCs have innate therapeutic effects by virtue of their miRNA cargoes.^[^ ^7a^ ^]^ Here, we profiled miRNAs within EVs and found that they were mainly enriched in pathways of cell death and proliferation, angiogenesis, and fibrosis, explaining the nephroprotective functions of the PBP‐EVs we observed. AKI, as a systematic process involving multiple interactions among injured tubules, inflammatory cells, endothelial cells, and fibroblasts, is highly regulated by complicated signaling networks, which suggests that targeting a certain kind of cell to regulate a single pathogenic process might not be sufficient to protect against AKI in simultaneously occurring processes.^[^ ^14^ ^]^ We have reason to believe that holistic treatment, such as EVs carrying a variety of therapeutic molecules, is the only reasonable strategy to protect against AKI owing to the simultaneous contributions of multiple interactions and the potential for single functional molecules to differentially regulate key mechanisms of different pathogenic processes.

In addition to their inherent functional cargoes, PBP‐EVs can carry and transfer exogenous therapeutic agents to recipient cells, which is another attractive feature for their use as carriers in EC‐targeted AKI therapy. To date, EVs have been demonstrated to be efficient carriers for delivering multiple cargoes, such as proteins, nucleic acids, and small molecule drugs.^[^ ^15^ ^]^ Unlike existing liposomes or nanoparticles, EVs as carriers have several outstanding advantages, including less immunogenicity, extended blood half‐life, and low long‐term safety risk, which is profited from their natural lipid and surface protein composition.^[^ ^8^, ^12^ ^]^ In addition, numerous exogenous loading methods, such as simple incubation, chemical reaction, electroporation, and sonication, are feasible and valid for the packaging of cargoes into EVs ex vitro.^[^ ^16^ ^]^ Thus, the attraction of PBP‐EVs developed in our study is that, in addition to spatiotemporal monitoring and EC targeted delivery, they can load exogenous drugs simultaneously, providing a practical AKI theranostic platform for multiple drug delivery.

The PBP‐EVs we designed in this study integrated real‐time monitoring and effective treatment and provided spatiotemporal information to help guide therapy while protecting against kidney injury. These features would facilitate their clinical applications in personalized therapy for AKI, resulting in optimal long‐term outcomes with reduced treatment time and cost. Clearly, PBP‐EVs hold great promise in establishing a theranostic system for the early diagnosis and personalized medicine of AKI and promoting its long‐term outcomes. EV‐based theranostics, in close combination with bioinspired materials and gene therapy, we believe will become a critical strategy in precision and personalized medicine, and the transformation of EV‐based theranostics from basic research into the clinic is likely to be achieved in the near future.

## 4 Experimental Section

### Mice and Animal Models

In this study, C57BL/6 mice (male, 8–10 weeks old) were purchased from the Laboratory Animal Center of the Academy of Military Medical Science (Beijing, China). For angiogenesis imaging, Vegfr2‐Fluc‐KI transgenic mice on a C57BL/6 albino and outbred (Nu/Nu) background were obtained from Xenogen Corporation (Hopkinton, MA). In Vegfr2‐Fluc‐KI mice, firefly luciferase (Fluc) was expressed under the promoter of vascular endothelial growth factor receptor 2 (Vegfr2) to report angiogenesis in vivo.^[^ ^17^ ^]^ All the animal treatments and the experimental procedures of the present study were approved by the Animal Experiments Ethical Committee of Nankai University and were in accordance with the NIH Guide for the Care and Use of Laboratory Animals (approval no. 20 190 022). Mice were housed in the specific pathogen‐free housing facility at a constant temperature (21–23 °C) and humidity (45–50%) in a 12 h light‐dark cycle (lights on 07:00–19:00 h), with food and water available ad libitum throughout the studies.

The renal ischemia/reperfusion injury (IRI) models were established as previously described.^[^ ^18^ ^]^ Briefly, mice were anesthetized by intraperitoneal injection of avertin (2.5%, 240 mg kg^−1^, Sigma‐Aldrich, Oakville, ON, Canada), and their eyes were covered with ophthalmic ointment to prevent dryness. To establish different degrees of renal damage models, the left renal pedicle was clamped for 15, 30, and 45 min and then released to allow blood reperfusion, which was used to induce mild, moderate, and severe renal IRI, respectively. Reperfusion was visually confirmed before the incision was closed. After 12 h of reperfusion, 100 µg of EVs or PBP‐EVs were injected intravenously at a total volume of 200 µL.

### Cells and EVs

Human placenta‐derived mesenchymal stem cells (hP‐MSCs) were isolated from placental tissues that were digested by collagenase II and trypsin as previously described.^[^ ^19^ ^]^ Isolated cells were cultured in Dulbecco's modified Eagle medium/nutrient mixture F‐12 (DMEM/F12) medium (Gibco, Grand Island, NY) supplemented with fetal bovine serum (FBS; 10%, Gibco), penicillin (100 U mL^−1^, Gibco), and streptomycin (100 µg mL^−1^, Gibco). In this study, hP‐MSCs at passages 4–10 were used. The endothelial cells (ECs) used in this study were human umbilical vein endothelial cells (HUVECs) obtained from the American Type Culture Collection (ATCC; Manassas, VA). HUVECs were cultured in endothelial cell growth medium‐2 (EGM2; Lonza, Walkersville, MD) and used between passages 6 and 10. The tubular epithelial cells (TECs) used in this study were HK2 obtained from ATCC and cultured in serum‐free keratinocyte medium (K‐SFM; Gibco) with 0.05 mg mL^−1^ bovine pituitary extract (BPE), 5 ng mL^−1^ human recombinant epidermal growth factor (EGF), and 100 units mL^−1^ penicillin–streptomycin. All cells were maintained in a humidified incubator (Thermo Scientific, Madison, WI) with 5% CO_2_ at 37 °C. To mimic the microenvironment in kidneys with severe IRI, hypoxia/reoxygenation (H/R) was performed on HUVECs or HK2 cells. Cells were incubated in a humidified hypoxia incubator (Thermo Scientific) with 5% CO_2_, 1% O_2_, and 94% N_2_ at 37 °C for 6 h and then reoxygenated with 95% air and 5% CO_2_ for an additional 12 h, as previously reported.^[^ ^20^ ^]^

EVs used in this study were isolated from the hP‐MSC supernatant as previously described.^[^ ^21^ ^]^ Briefly, hP‐MSCs were continuously passaged every 2 days with DMEM/F12 medium containing 10% EV‐free FBS, penicillin (100 U mL^−1^, Gibco), and streptomycin (100 µg mL^−1^, Gibco). EV‐free FBS was obtained by ultracentrifuging FBS at 100 000 × g for 18 h. The supernatant was collected during subculture and centrifuged at 500 × g for 10 min, 2000 × g for 30 min, and 10 000 × g for 30 min in sequence to remove any cell debris and apoptotic bodies. The EVs were collected by ultracentrifugation at 100 000 × g for 70 min and washed with phosphate‐buffered saline (PBS) by a second ultracentrifugation step at 100 000 × g for 2 h. Finally, the precipitated EVs were resuspended in 200 µL PBS and stored in a −80 °C refrigerator. All steps were performed at 4 °C under aseptic conditions. EVs were quantified by the total amount of protein using a bicinchoninic acid (BCA) protein assay kit (Thermo Scientific).

### Fabrication of PBP‐EVs

Cy5.5‐labeled DPP was dissolved in PBS at a final stocking concentration of 500 µm. To optimize the preparation conditions of PBP‐EVs, different final molar concentrations (1, 2, 5, and 10 µm) of Cy5.5‐labeled DPP were incubated with EVs at 4, 25, and 37 °C for different times (10, 20, 30, and 60 min). Then, 5 volumes of PBS was used to wash out the excess Cy5.5‐labeled DPP for more than 5 times by ultrafiltration (100 kDa ultrafiltration centrifugal tube) until the PBP‐EVs were concentrated to the initial concentration. The modification efficiency of Cy5.5‐labeled DPP for EVs was assessed according to the Cy5.5 fluorescence intensity by flow cytometry analysis. According to the optimal modification conditions, the PBP‐EVs were fabricated by coincubating EVs with 5 µm DPP at room temperature (25 °C) for 30 min followed by ultrafiltration to remove excess DPP.

### Flow Cytometry Analysis of PBP‐EVs

EVs and PBP‐EVs were analyzed using a FACSCalibur flow cytometer (FCM; BD Biosciences, San Jose, CA) as previously reported.^[^ ^22^ ^]^ Briefly, the PBS and DPP solutions were first tested to evaluate the background noise. The unmodified EVs were used to set suitable voltages and thresholds and gate the EV population in the forward‐scatter channel and side‐scatter channel for measurements. To determine the best modification conditions of DPP‐labeled Cy5.5 for EVs, the different molarities of Cy5.5‐labeled DPP were incubated with EVs at 4, 25, and 37 °C for different times, followed by filtration with 100 kDa ultrafiltration centrifugal tubes to remove excess DPP before FCM. To assess the stability of PBP‐EVs, PBP‐EVs were preserved undisturbed at 4 °C for 1, 3, and 7 days and then analyzed by FCM. Data were analyzed using FlowJo software (Tree Star, San Carlos, CA).

### Characterization of PBP‐EVs

The size distribution and zeta potential of EVs and PBP‐EVs were determined using a Malvern Particle Size Analyzer (Zeta sizer Nano ZS, Malvern Panalytical, Malvern, UK). Then, the morphologies of EVs and PBP‐EVs were observed using transmission electron microscopy (TEM; Talos L120C, Hillsboro, OR) as previously reported. In summary, 2 µg µL^−1^ EVs or PBP‐EVs were loaded in a carbon film (Zhongjingkeji Technology, Beijing, China) with negative staining with 2% phosphotungstic acid. The samples were allowed to air dry and then observed using TEM at an acceleration voltage of 120 kV. Finally, the three EV marker proteins (Alix, TSG101, and CD63) were analyzed by Western blotting.

### Multimodal Imaging of PBP‐EVs

EVs labeled with Gaussia luciferase (Gluc) were harvested as previously described.^[^ ^23^ ^]^ Briefly, a gene fragment expressing the Gluc‐lactadherin fusion protein was transduced into hP‐MSCs, which allowed the EVs derived from hP‐MSCs to be labeled with Gluc. The hP‐MSCs were infected with lentivirus particles (MOI = 10) that expressed a Gluc and human lactadherin fusion protein and then screened with puromycin (2 µg mL^−1^, Sigma–Aldrich). The Gluc‐labeled EVs were isolated from the supernatant of the infected hP‐MSCs by ultracentrifugation. The Gluc‐labeled EVs were incubated with DMPE‐PEG‐Cy5.5 and Cy5.5‐labeled DPP to obtain Cy5.5/Gluc‐labeled EVs and Cy5.5/Gluc‐labeled PBP‐EVs, respectively. The Gluc radiance of EVs or PBP‐EVs was monitored by bioluminescence imaging using an IVIS Lumina Imaging System (Xenogen Corporation). For in vitro experiments, Gluc‐labeled EVs or PBP‐EVs were detected in 24‐well plates with 0.1 µg mL^−1^ water‐soluble coelenterazine (CTZ; Nanolight Technology, Pinetop, AZ) as substrate. Regarding in vivo experiments, 100 µg of Gluc‐labeled EVs or PBP‐EVs were intravenously injected through the caudal vein. At the indicated time points, mice were imaged immediately after intraperitoneal injection of water‐soluble CTZ (5 mg kg^−1^; Nanolight Technology) for bioluminescence imaging. All the Gluc radiance was measured by the average radiance from regions of interest. The Cy5.5 radiance of EVs or PBP‐EVs was monitored by fluorescence imaging using an IVIS Lumina Imaging System (excitation, 670 nm; emission, 694 nm) and measured by the radiant efficiency of regions of interest. The Cy5.5 radiance of EVs or PBP‐EVs in cells and renal sections was observed by a laser scanning confocal microscope (LSCM; excitation, 647 nm; FV1000, Olympus, Lake Success, NY) and measured by fluorescence intensity from regions of interest.

### Kidney Targeting and Biodistribution of PBP‐EVs

Renal IRI mice were randomly divided into two groups and injected intravenously with Cy5.5/Gluc‐labeled EVs or PBP‐EVs (100 µg per mouse). At the indicated time point post injection, the Gluc radiance of the mice was captured using the IVIS Lumina Imaging System. To investigate the biodistribution of PBP‐EVs in renal IRI mice, Cy5.5 signals from EVs and PBP‐EVs from kidneys and other organs were examined using the IVIS Lumina Imaging System and LSCM. At the indicated time point postinjection, perfusion was performed with 40 mL of cold PBS to wash out the EVs or PBP‐EVs in circulation. The injured kidneys and other major organs were harvested for Cy5.5 imaging at excitation/emission wavelengths of 670/694 nm. To further study the in vivo fate and cellular distribution of the injected PBP‐EVs, the collected kidneys were fixed and sectioned into 5 µm cryosections for immunostaining and observed by LSCM.

### Bioluminescence Imaging of Renal Angiogenesis

The angiogenesis of the injured kidney was examined in Vegfr2‐Fluc KI transgenic mice by bioluminescence imaging as mentioned before.^[^ ^24^ ^]^ Cy5.5/Gluc‐labeled EVs and PBP‐EVs (100 µg per mouse) were injected intravenously 12 h postsevere IRI. Mice injected with an equal volume of PBS served as controls. At the indicated time points, mice were intraperitoneally injected with D‐luciferin (150 mg kg^−1^; Biosynth International, Naperville, IL) and then imaged within the IVIS Lumina Imaging System. Fluc signals were measured by the average radiance from regions of interest using Living Image software.

### Cell Cycle Assay

HK2 cells used for the cell cycle assay were cultured in the lower chamber of the modified Transwell system (3452, Corning) and subjected to H/R. EVs or PBP‐EVs (100 µg mL^−1^) were added to the upper chamber postreoxygenation and cultured for another 24 h. Single kidney cells were isolated at day 3 postsevere IRI using the multitissue dissociation kit 2 (#130‐110‐203, Miltenyi Biotec, Bergisch Gladbach, Germany). In summary, the quarter kidneys were cut and dissociated by an enzyme mixture at 37 °C for 35 min. The cell precipitates were screened using a 30 µm filter to obtain renal single cells. HK2 or collected renal cells were fixed with 75% ethanol at 4 °C overnight. The fixed cells were stained with propidium iodide (PI) using a Cell Cycle and Apoptosis Analysis Kit (Beyotime Biotechnology, Shanghai, China) at 37 °C for 15 min. The fractions of the stained cells in different phases of the cell cycle were detected by FCM and analyzed by FlowJo software.

### Renal Function Analysis

To assess the residual function of the injured kidney, unilateral (left) severe renal ischemia/reperfusion injury plus contralateral nephrectomy was performed in the animals. On days 3 and 7 postinjury, serum samples were collected to evaluate renal function markers, including blood urea nitrogen (BUN) and serum creatinine (SCr). The concentrations of BUN and SCr were measured by using a BUN assay kit (Nanjing Jiancheng Bioengineering Institute) and an SCr assay kit (Nanjing Jiancheng Bioengineering Institute), respectively.

### Histopathology and Immunostaining

Mice were euthanized to harvest the tissue samples at the indicated time points. Tissue samples were fixed in 4% paraformaldehyde, dehydrated with gradient ethanol, hyalinized with xylene, embedded in paraffin, and cut into 5 µm paraffin sections. For cryosectioning, tissue samples were fixed with 4% paraformaldehyde, dehydrated with a 30% sucrose solution, embedded in Optimal Cutting Temperature (OCT) compound, and sectioned to a thickness of 5 µm. Hematoxylin and eosin (H&E) staining and Masson's trichrome staining were performed with paraffin sections according to a standard protocol.

For immunohistochemical staining, paraffin sections were antigen repaired and then incubated with primary antibodies against Kim1 (1:200; ab47635, Abcam), CD45 (1:50; 550 539, BD Transduction Laboratories, Franklin Lake, NJ) or Caspase3 (1:200; WL02117, Wanleibio) and then analyzed using the horseradish peroxidase streptavidin detection system (ZSGB‐BIO, Beijing, China) according to the manufacturer's protocol. After counterstaining with hematoxylin, the immunoreactivity was visualized using diaminobenzidine (DAB, ZSGB‐BIO). For immunofluorescence staining, cryosections were incubated with primary antibodies against P‐selectin (1:100; sc8419, Santa Cruz Biotechnology), CD31 (1:200; 550 274, BD Transduction Laboratories), E‐cadherin (1:200; ab76055, Abcam), *α*‐SMA (1:200; ab7817, Abcam), F4/80 (1:50; ab16911, Abcam), Ki67 (1:200; ab16667, Abcam), Kim1 (1:200; ab47635, Abcam), and Collagen IV (1:200; ab6586, Abcam), followed by incubation with secondary antibodies labeled Alexa Fluor 488, Alexa Fluor 594, or Alexa Fluor 647 (Life Technologies, Carlsbad, CA). Lotus tetragonolobus lectin labeled with FITC (LTL, 1:400; Vector Laboratories) was used to reveal the renal structure, and DAPI (1:2000) was used to visualize the nucleus.

Immunocytochemistry was conducted as previously described.^[^ ^25^ ^]^ Briefly, cells cultured on glass coverslips were fixed in 4% paraformaldehyde and then washed twice with PBS. After blocking with 10% goat serum, cell samples were incubated with primary antibodies against P‐selectin (1:100; sc8419, Santa Cruz Biotechnology), Ki67 (1:200; 550 609, BD Transduction Laboratories), and p‐H3 Ser10 (1:200; #9701, Cell Signaling Technology, Danvers, MA), followed by secondary antibodies labeled Alexa Fluor 488 or Alexa Flour 594. Alexa Fluor 488‐labeled phalloidin (1:400; Invitrogen) was used to visualize cellular morphology by binding to actin. Cell samples were finally mounted on DAPI‐Fluoromount G (Vector Laboratories). Images were visualized under an LSCM. All data were analyzed by ImageJ software.

### Statistical Analysis

All studies were evaluated in at least three independent experiments for each condition to ensure reproducibility. Data are expressed as scatter plots with the mean ± s.d. Significant differences between different groups were determined using two‐tailed unpaired Student's *t*‐tests for two‐group comparisons and one‐ or two‐way analysis of variance (ANOVA) with post hoc Tukey's test for multiple group comparisons. Statistical analyses were performed using GraphPad Prism software. Differences were considered statistically significant at *P* < 0.05.

## Conflict of Interest

The authors declare no conflict of interest.

## Author Contributions

Z.L., X.M.C., and K.Z. conceived and designed the experiments and revised the manuscript. K.Z. and R.L. performed the experiments. D.K., K.W., and H.Y. contributed to the design and synthesis of DPP. X.Z., H.H., and Y.L. bred and identified the transgenic animals. Z.K., X.C., and S.C. analyzed the data. Z.C.H. and Z.H. provided technical support. K.Z. and Z.L. wrote the manuscript, and all authors approved the final version of this manuscript.

## Supporting information

Supporting Information

## Data Availability

The data that support the findings of this study are available from the corresponding author upon reasonable request.
