# Supplementary material for: Renal Endothelial Cell‐Targeted Extracellular Vesicles Protect the Kidney from Ischemic Injury
Source: Adv Sci (Weinh). 2022 Nov 23;10(3):2204626. doi: 10.1002/advs.202204626 (PMC9875634; doi:10.1002/advs.202204626)
Supplement: Supplementary file 1 — Supporting Information [file ADVS-10-2204626-s001.pdf]

ADVANCED SCIENCE
Open Access

Supporting Information

for *Adv. Sci.*, DOI 10.1002/advs.202204626

Renal Endothelial Cell-Targeted Extracellular Vesicles Protect the Kidney from Ischemic Injury

*Kaiyue Zhang, Rongrong Li, Xiaoniao Chen, Hongyu Yan, Huifang Li, Xiaotong Zhao, Haoyan Huang, Shang Chen, Yue Liu, Kai Wang, Zhibo Han, Zhong-Chao Han, Deling Kong, Xiang-Mei Chen and Zongjin Li**

Supplemental Information

Supplementary figures and legends

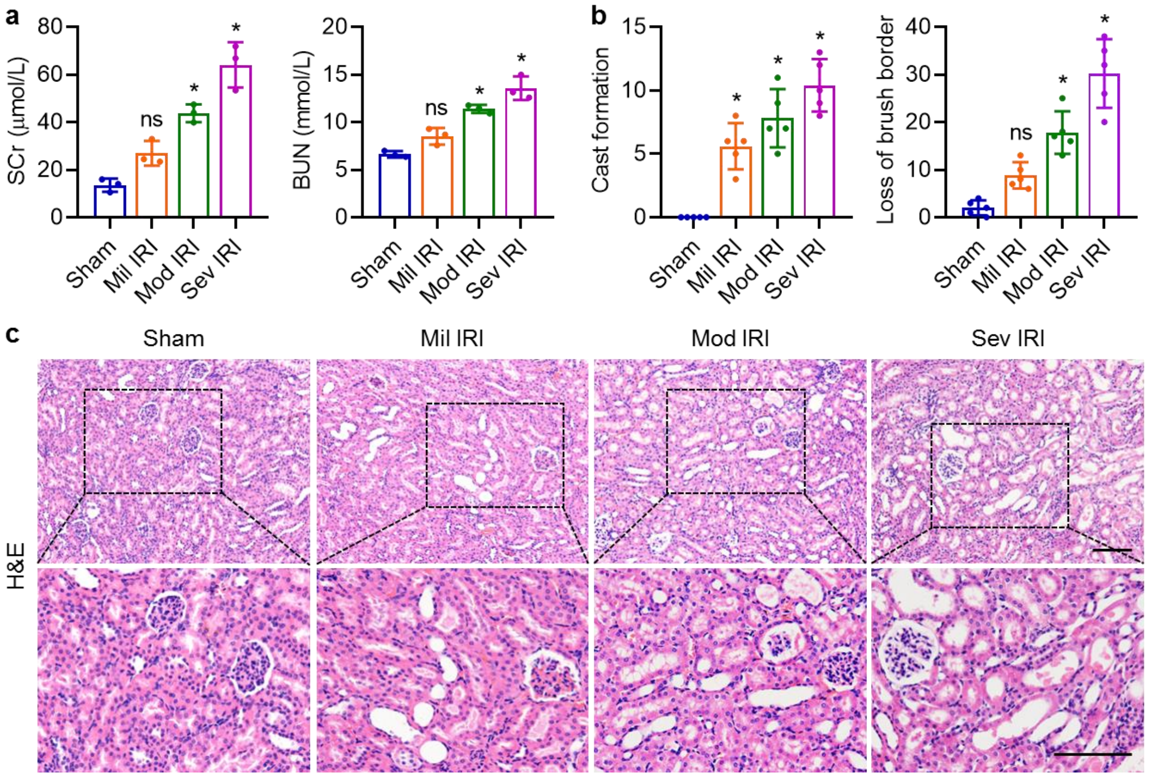

Supplementary Fig. 1 | Evaluations of renal function and structure post mild, moderate, and severe IRI. a, Serum creatinine (SCr) and blood urea nitrogen (BUN) were evaluated on day 3 post mild, moderate, and severe IRI. n = 3. **b**, Quantification of cast formation and loss of brush border in H&E staining images. n = 5. **c**, H&E staining of renal tissues 3 days post mild, moderate, and severe IRI. Scale bar, 100 μm. All data are expressed as mean ± s.d. Statistical analysis was performed using one-way ANOVA with Tukey's multiple comparison tests. ns, not significant versus Sham, *$P < 0.05$ versus Sham.

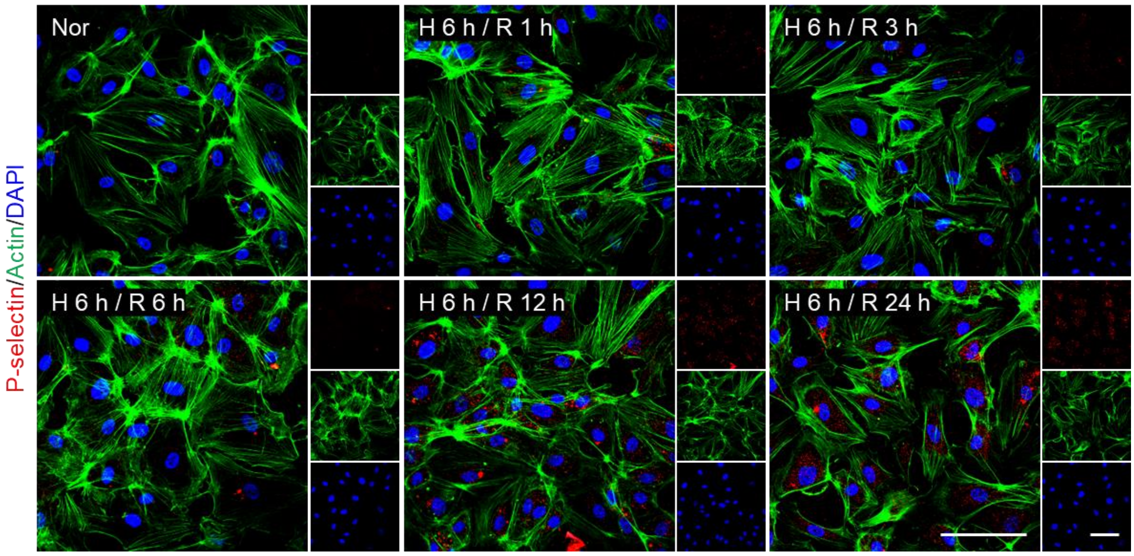

Supplementary Fig. 2 | Expression of P-selectin in H/R injured HUVECs. P-selectin (red) expression in HUVECs (Alexa Fluor 488-labeled phalloidin, green, actin) after hypoxia for 6 h and reoxygenation for different times was revealed by immunofluorescence. The nuclei were counterstained with DAPI (blue). Scale bars, 100 μm.

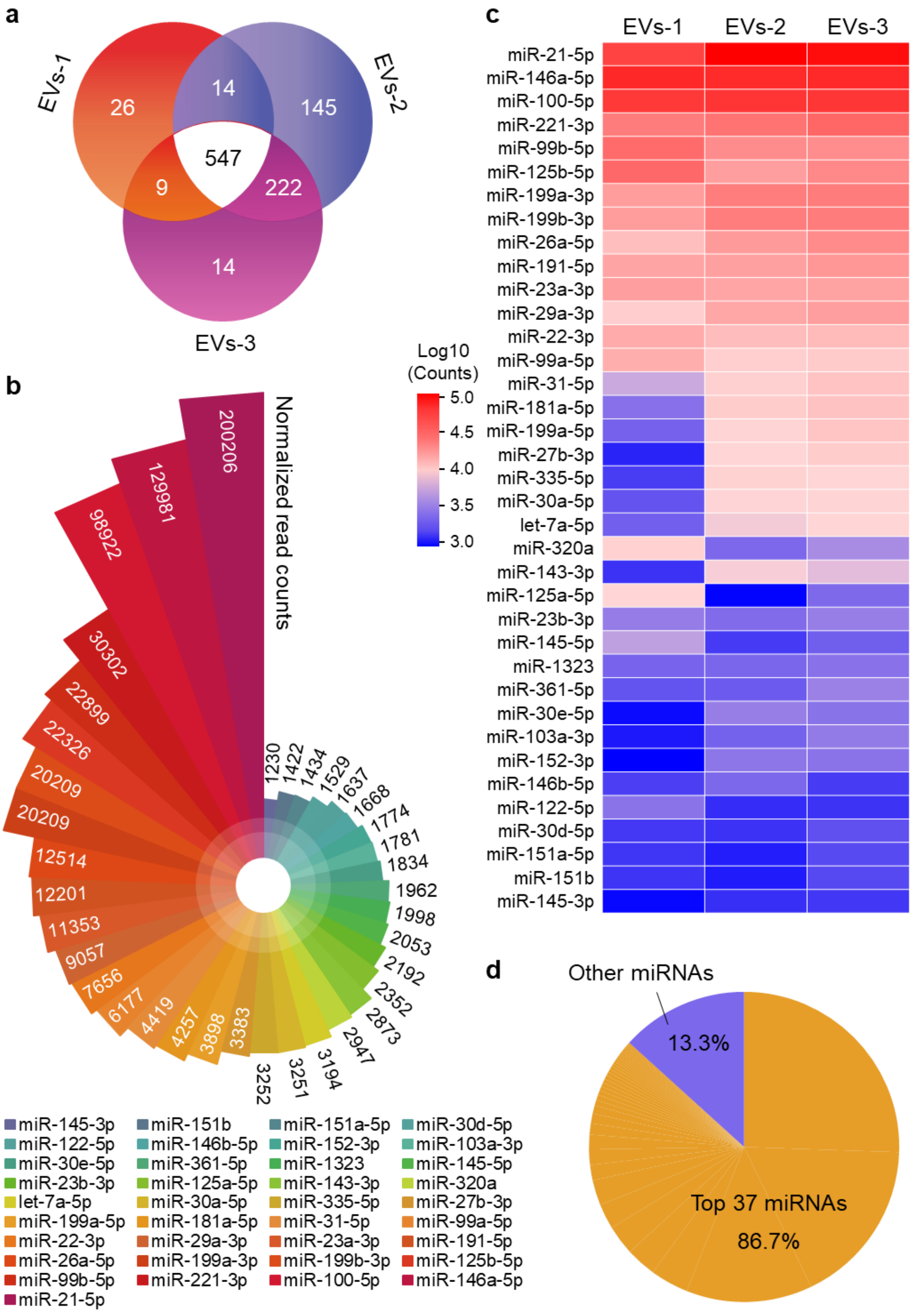

Supplementary Fig. 3 | Profile of miRNAs within MSC derived EVs. a Venn diagram of all miRNAs identified in EVs derived from three donors. **b**, Nightingale's rose diagram showed the average reads of the top 37 miRNAs in EVs derived from three donors. **c**, Heatmap of the top 37 miRNAs in EVs derived from three donors. **d**, The top 37 miRNAs

accounted for 86.7% of the total miRNAs present in EVs.

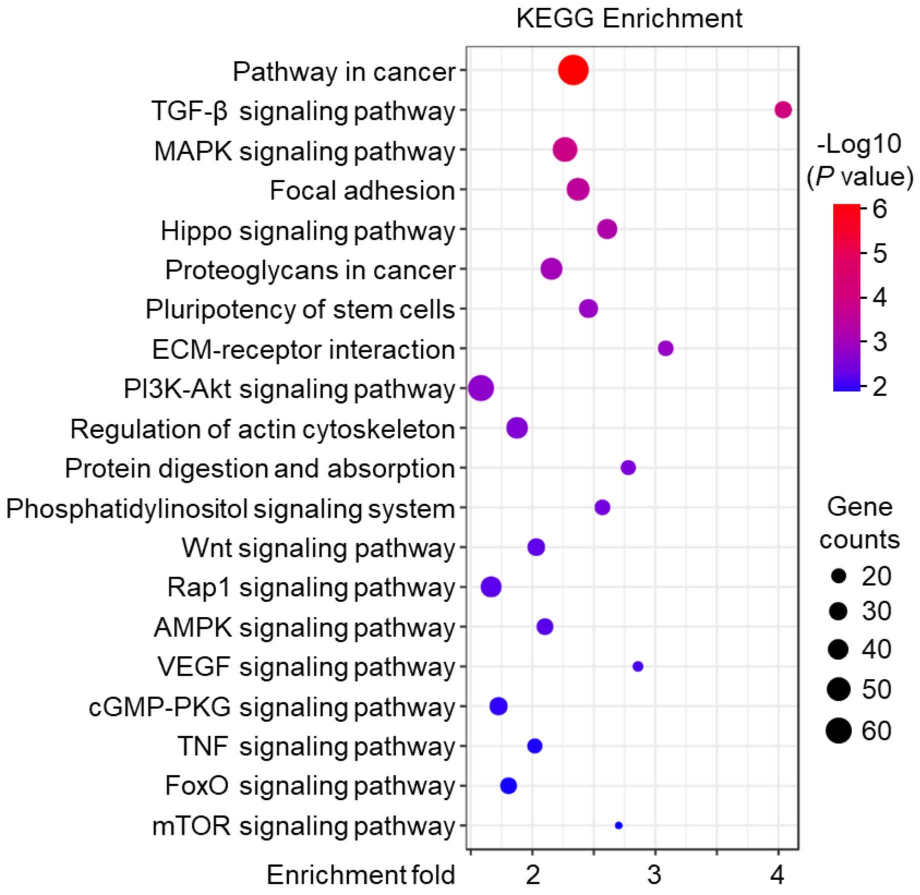

Supplementary Fig. 4 | KEGG enrichment of the top 37 miRNAs targeted. Bubble plot of enriched pathways targeted by the top 37 miRNAs ranked according to the *P*-value from high to low.

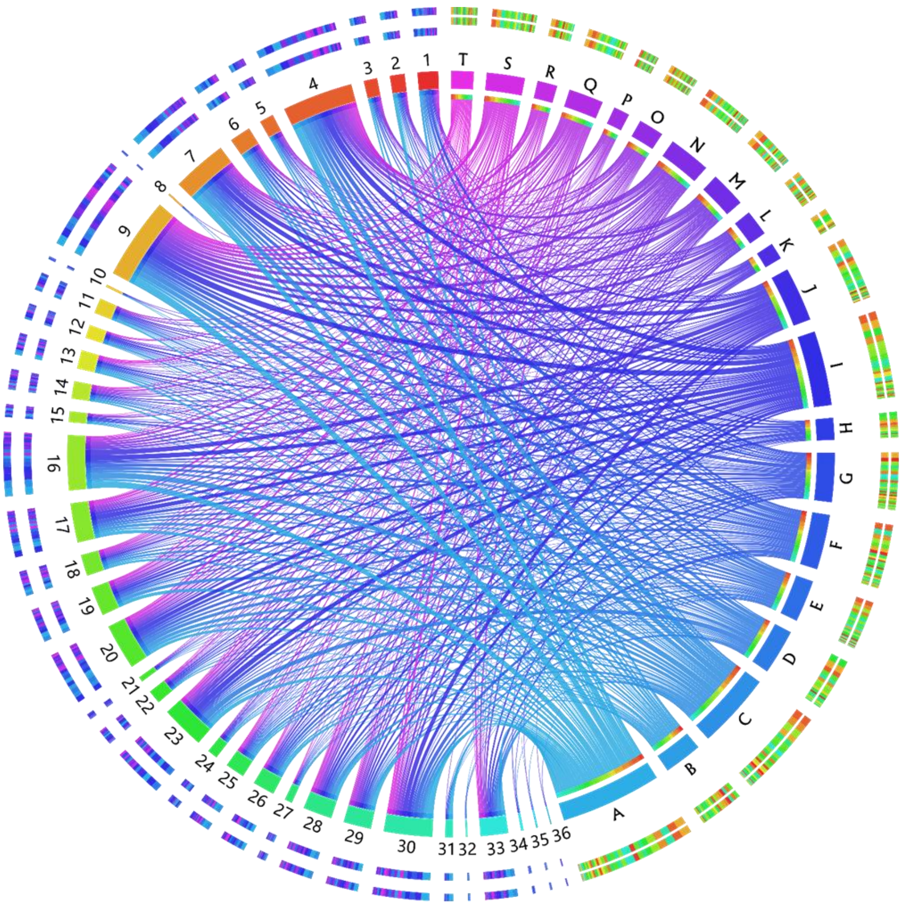

1 miR-21-5p	7 miR-199a-3p	13 miR-22-3p	19 miR-30a-5p	25 miR-145-5p	31 miR-146b-5p
2 miR-146a-5p	8 miR-199b-3p	14 miR-31-5p	20 let-7a-5p	26 miR-1323	32 miR-122-5p
3 miR-100-5p	9 miR-26a-5p	15 miR-181a-5p	21 miR-320a	27 miR-361-5p	33 miR-30d-5p
4 miR-221-3p	10 miR-191-5p	16 miR-199a-5p	22 miR-143-3p	28 miR-30e-5p	34 miR-151a-5p
5 miR-99b-5p	11 miR-23a-3p	17 miR-27b-3p	23 miR-125a-5p	29 miR-103a-3p	35 miR-151b
6 miR-125b-5p	12 miR-29a-3p	18 miR-335-5p	24 miR-23b-3p	30 miR-152-3p	36 miR-145-3p

A: Pathways in cancer B: TGF-β signaling pathway C: MAPK signaling pathway D: Focal adhesion
E: Hippo signaling pathway F: Proteoglycans in cancer G: Signaling pathways regulating pluripotency of stem cells
H: ECM-receptor interaction I: PI3K-Akt signaling pathway J: Regulation of actin cytoskeleton
K: Protein digestion and absorption L: Phosphatidylinositol signaling system M: Wnt signaling pathway
N: Rap1 signaling pathway O: AMPK signaling pathway P: VEGF signaling pathway
Q: cGMP-PKG signaling pathway R: TNF signaling pathway S: FoxO signaling pathway T: mTOR signaling pathway

Supplementary Fig. 5 | Chord diagram of the top 37 miRNAs and enriched pathways they targeted. The amounts of genes targeted by each miRNA in every enriched pathway were revealed in the chord diagram.

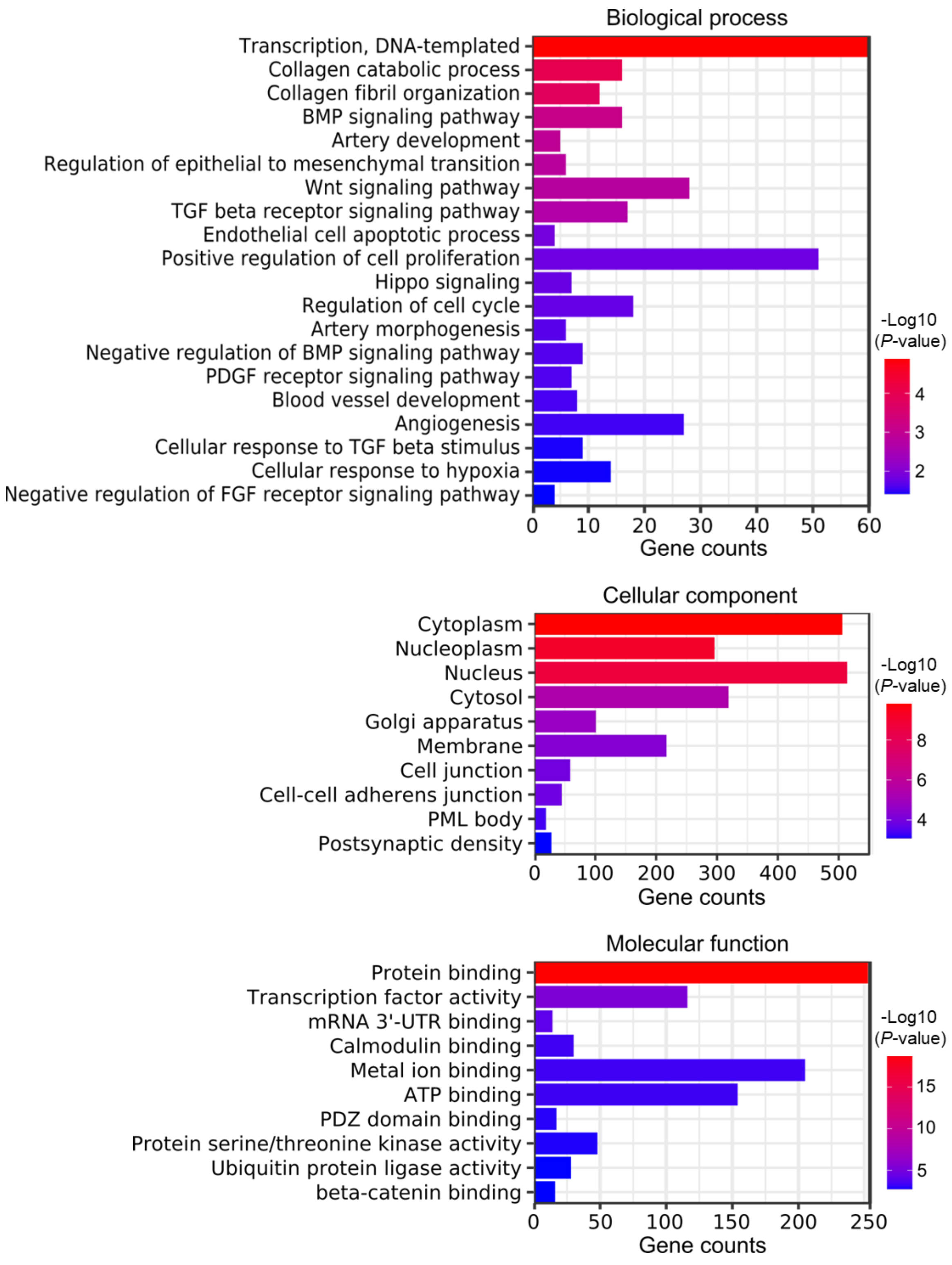

Supplementary Fig. 6 | Gene ontology (GO) enrichment analysis of target genes. Enriched biological processes, cellular components, and molecular functions targeted by the top 37 miRNAs ranked based on the *P*-value from high to low.

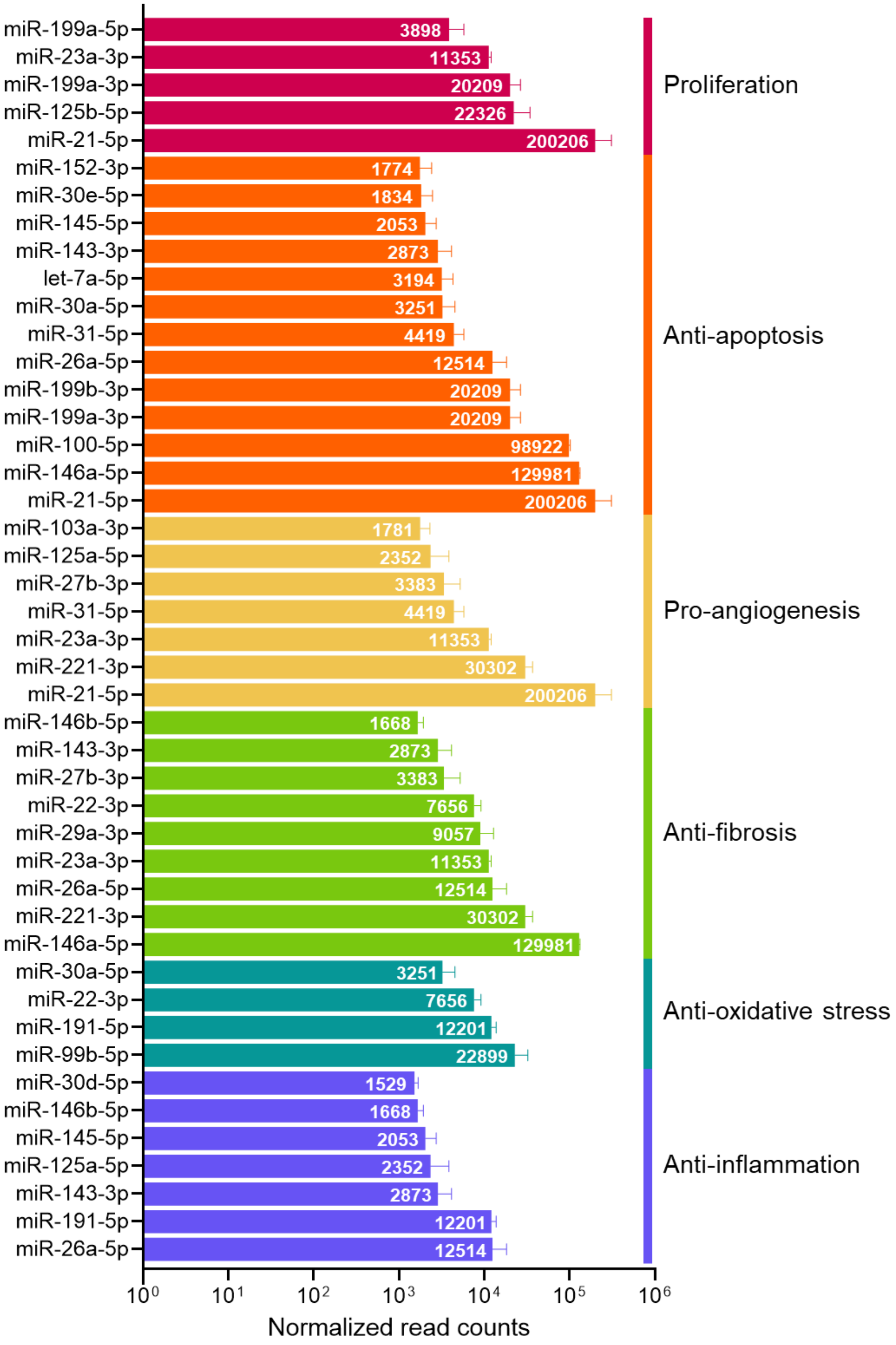

Supplementary Fig. 7 | Functional classification of the top 37 miRNAs. The top 37 miRNAs were classified into 6 categories according to their therapeutic functions, including proliferation, anti-apoptosis, pro-angiogenesis, anti-fibrosis, anti-oxidative stress, and anti-inflammatory. n = 3. All data are expressed as mean ± s.d.

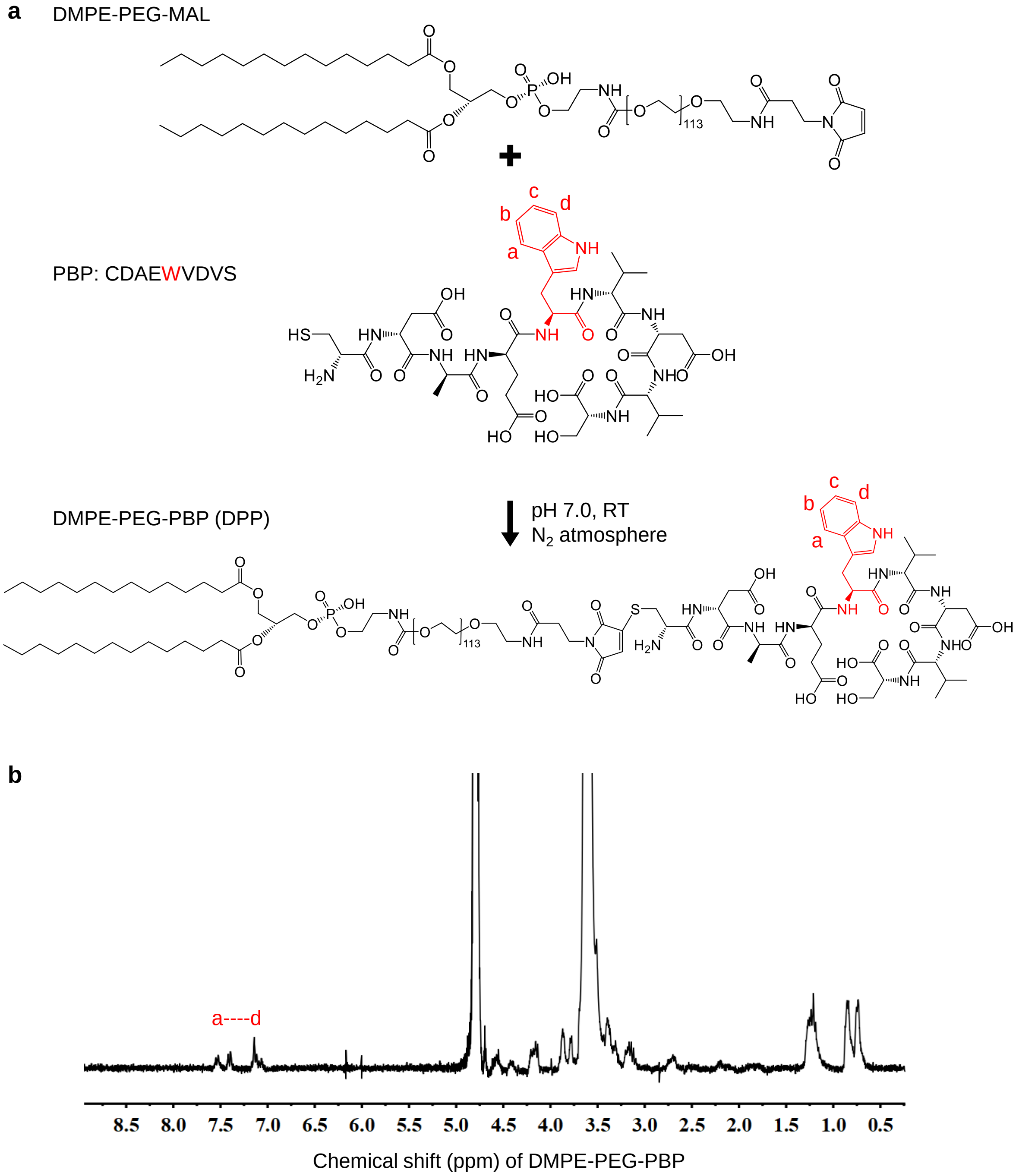

Supplementary Fig. 8 | Synthesis and identification of DMPE-PEG-BPB (DPP). a, Schematic illustration of the conjugating reaction between DMPE-PEG-Maleimide and Cysteine-terminated PBP. **b**, 1H-NMR spectra of DPP in D_2O.

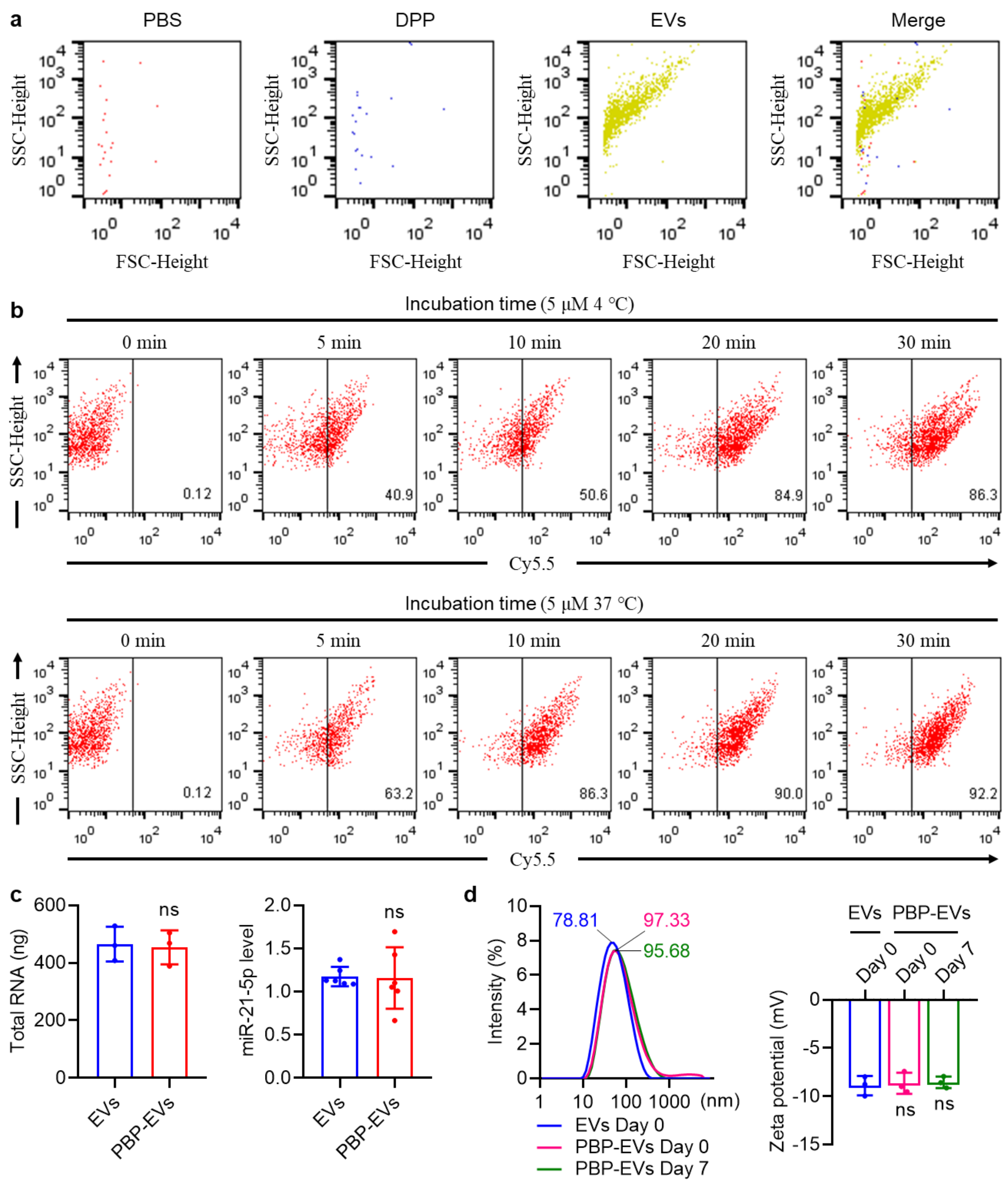

Supplementary Fig. 9 | Flow cytometry of EVs and PBP-EVs. **a**, Validation of flow cytometry for the measurement of EV. PBS and DPP solution (5 µM) were used to evaluate buffer background noise. **b**, Flow cytometry to assess the modification efficiency of Cy5.5-labeled DPP on EVs after incubation at 4 and 37 °C for different times. **c**, RNA change in EVs before and after DPP modification. **d**, Size distribution and zeta potential of PBP-EVs after storage at 4 °C for 7 days. All data are expressed as the mean ± s.d. For **c,** statistical analysis was performed using two-tailed unpaired Student's *t*-tests. For **d**, statistical analysis was performed using one-way ANOVA with Tukey's multiple comparison tests. ns, not significant versus EV.

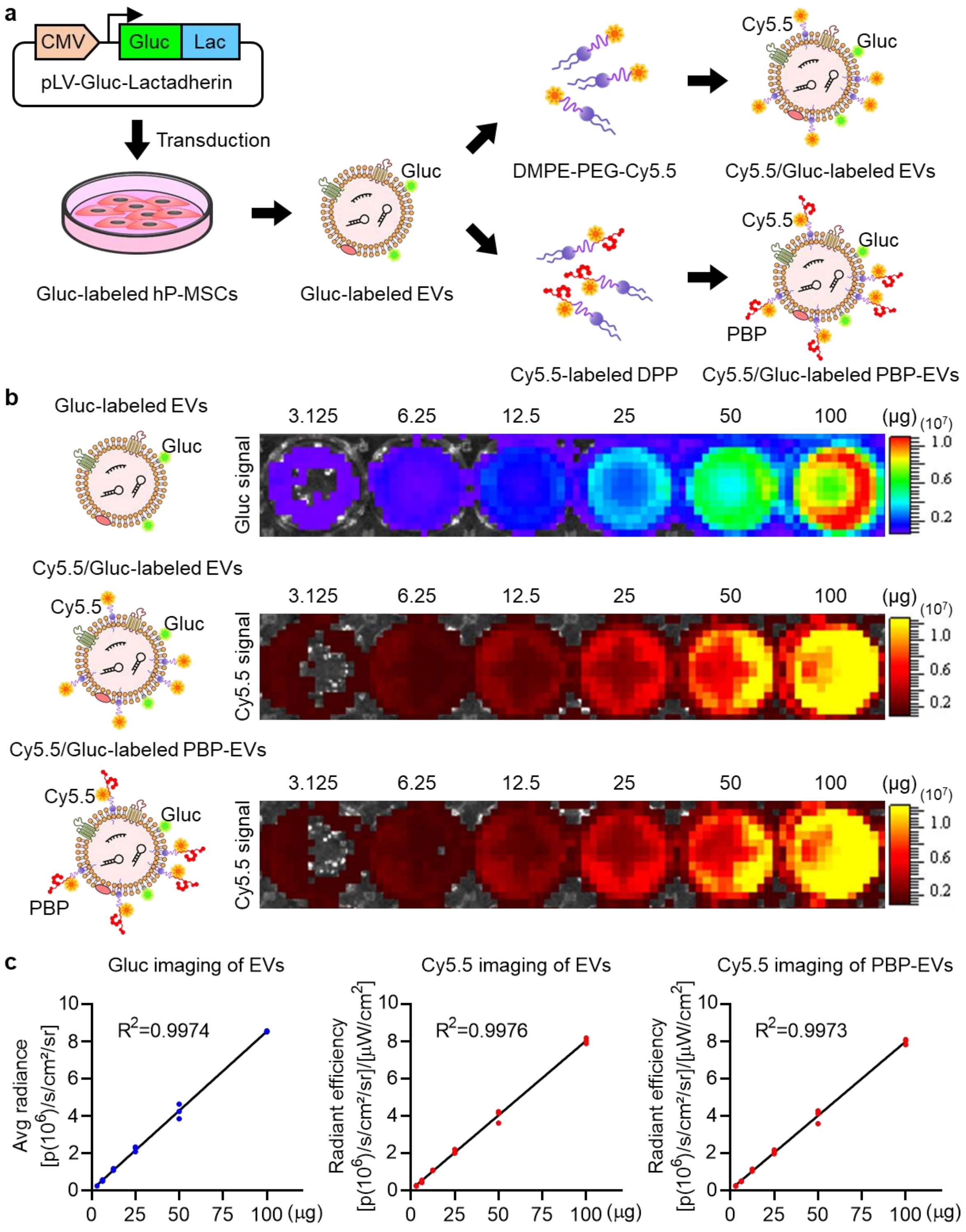

Supplementary Fig. 10 | Gluc and Cy5.5 imaging of EVs and PBP-EVs. a, Schematic diagrams of Gluc and Cy5.5 labeled EVs and PBP-EVs. **b**, Imaging of Gluc-labeled EVs, Cy5.5-labeled EVs, and Cy5.5-labeled PBP-EVs. **c**, Standard curve for Gluc imaging of Gluc-labeled EVs (R^2 = 0.9974), Cy5.5 imaging of Cy5.5-labeled EVs (R^2 = 0.9976), and Cy5.5 imaging of Cy5.5-labeled PBP-EVs (R^2 = 0.9973). The correlation between the

number of EVs (or PBP-EVs) and imaging signals was analyzed using linear regression. n = 3.

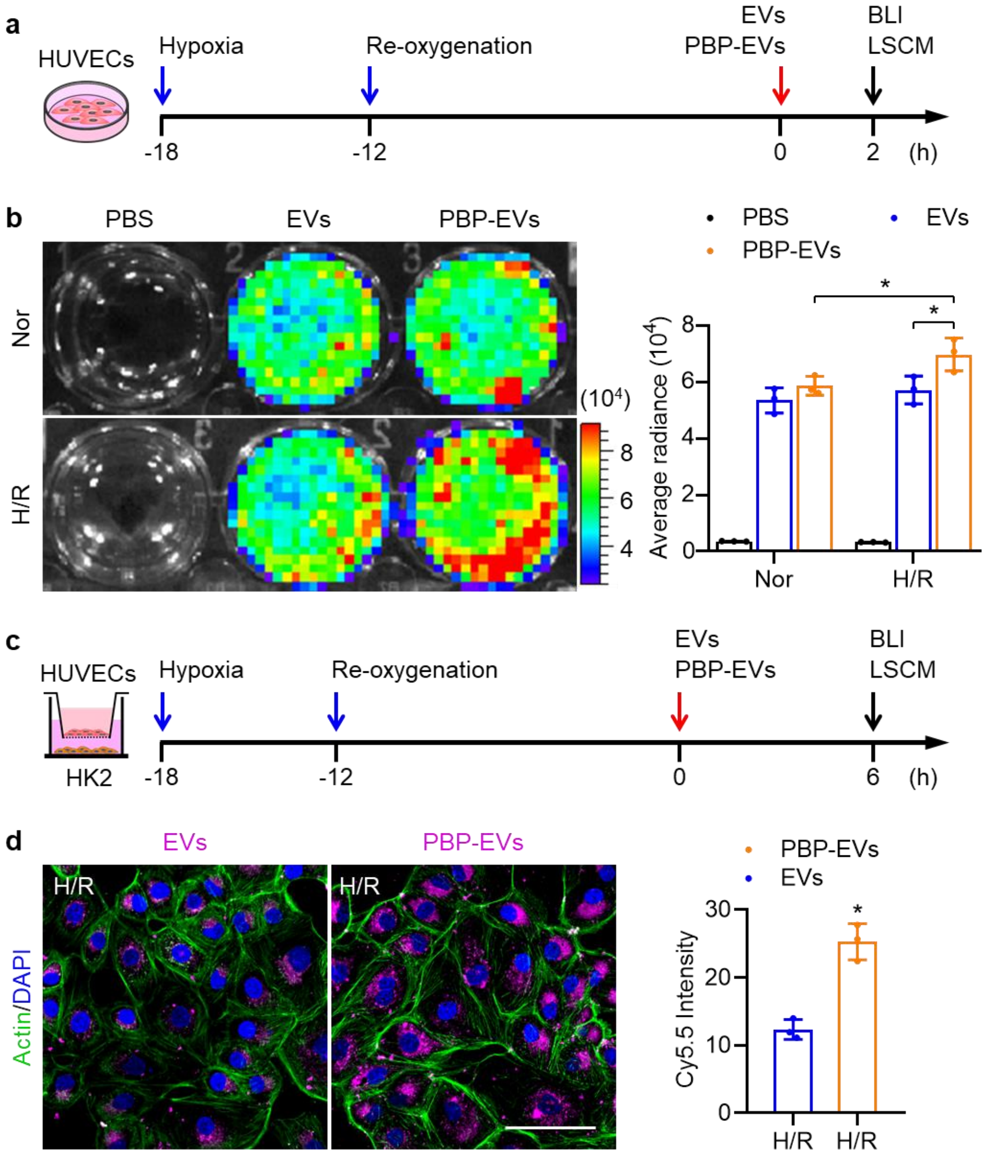

Supplementary Fig. 11 | Targeting ability of PBP-EVs *in vitro*. **a**, Schematic diagram showing study design of PBP-EVs targeting H/R injured HUVECs *in vitro*. **b**, Gluc imaging and quantification revealed H/R injured HUVECs internalized more PBP-EVs *in vitro*. n = 3. Statistical analysis was performed using two-way ANOVA with Tukey's multiple comparison tests. **c**, Schematic diagram showed the study design of PBP-EVs penetration of H/R injured endothelium *in vitro*. **d**, LSCM images and quantification showed an increased internalization of PBP-EVs (Cy5.5, yellow) in HK2 (Alexa Fluor 488-labeled phalloidin, green, actin) in lower chambers. The nuclei were counterstained with DAPI (blue). Scale bar, 100 μm. n = 3. Statistical analysis was performed using a two-tailed unpaired Student's *t*-test. All data are

expressed as mean ± s.d. *P < 0.05. BLI: bioluminescence imaging, LSCM: laser scanning confocal microscope.

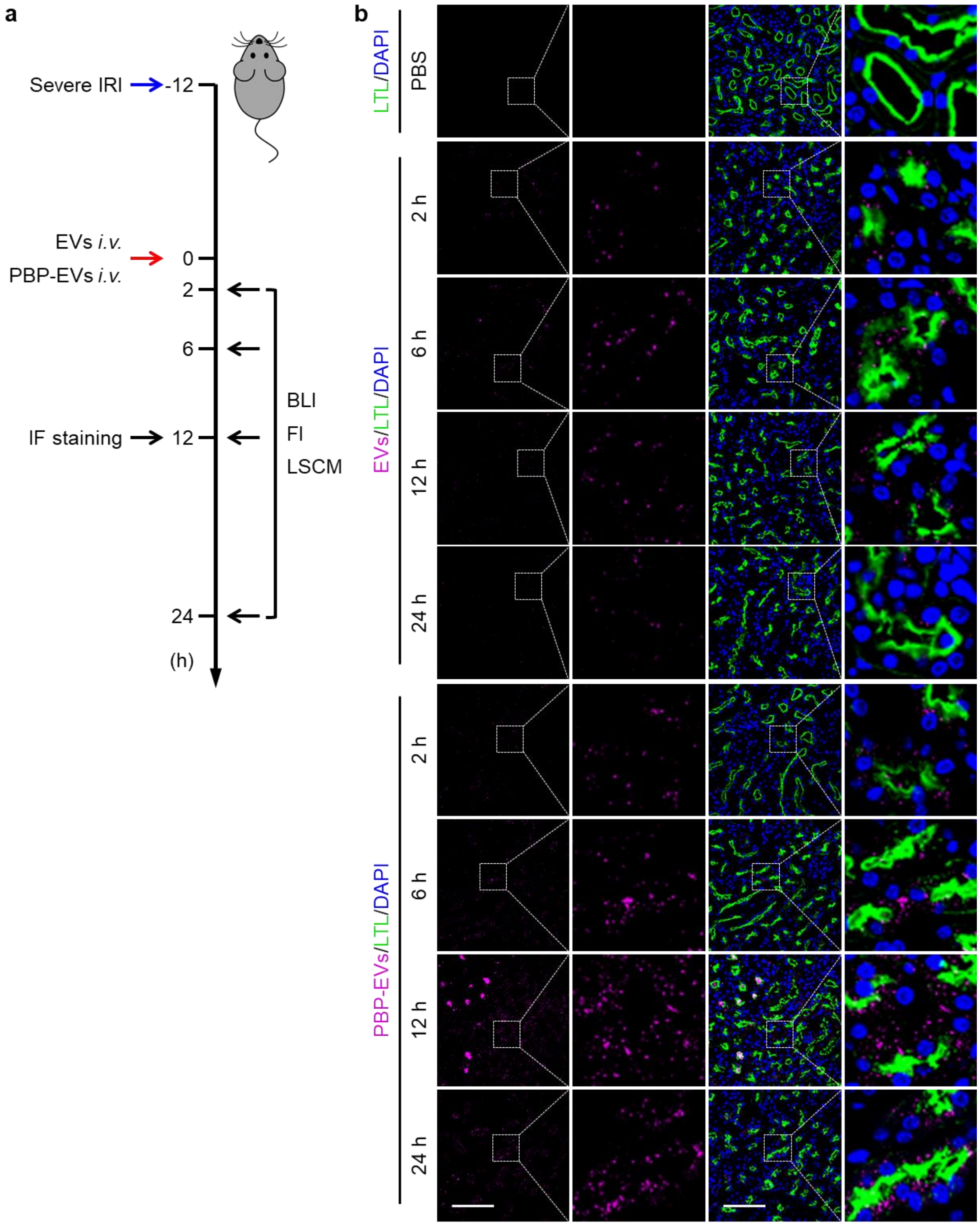

Supplementary Fig. 12 | Targeting ability of PBP-EVs *in vivo*. a, Schematic diagram revealed study design of PBP-EVs targeting injured kidneys *in vivo*. **b**, LSCM images and local zooms of EVs and PBP-EVs (Cy5.5, magenta) accumulated in injured renal tissues (FITC-labeled LTL, green, proximal tubules) at 2, 6, 12, and 24 h after injection. The nuclei were counterstained with DAPI (blue). Scale bar, 100 µm. BLI: bioluminescence imaging; FI: fluorescence imaging; LSCM: laser scanning confocal microscope.

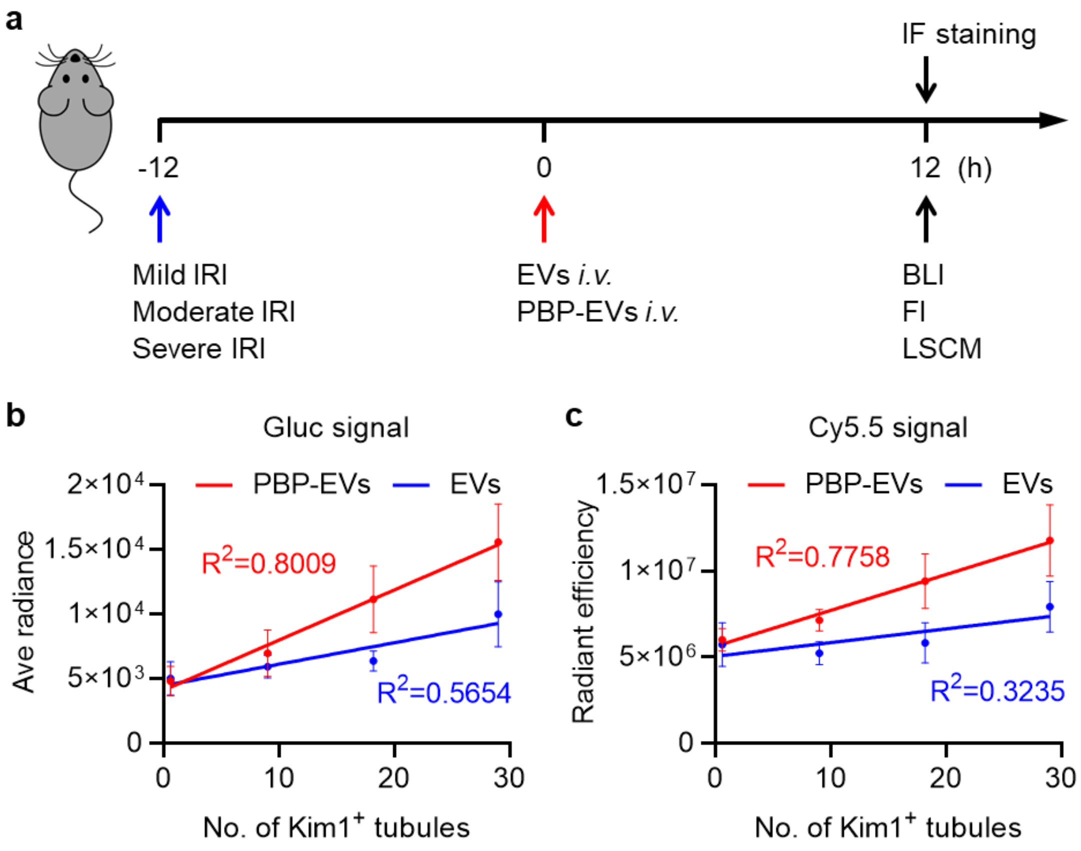

Supplementary Fig. 13 | Diagnostic potential of PBP-EVs *in vivo*. a, Schematic diagram of study design for PBP-EVs indicating the degree of renal injury. BLI: bioluminescence imaging; FI: fluorescence imaging; LSCM: laser scanning confocal microscope. **b**, Correlation between the number of Kim1$^+$ tubules in Fig 4f and Gluc signals in Fig 4b was analyzed using linear regression. **c**, Correlation between the number of Kim1$^+$ tubules in **Fig 4f** and Cy5.5 signals in Fig 4c was analyzed using linear regression. n = 5. All data are expressed as mean ± s.d.

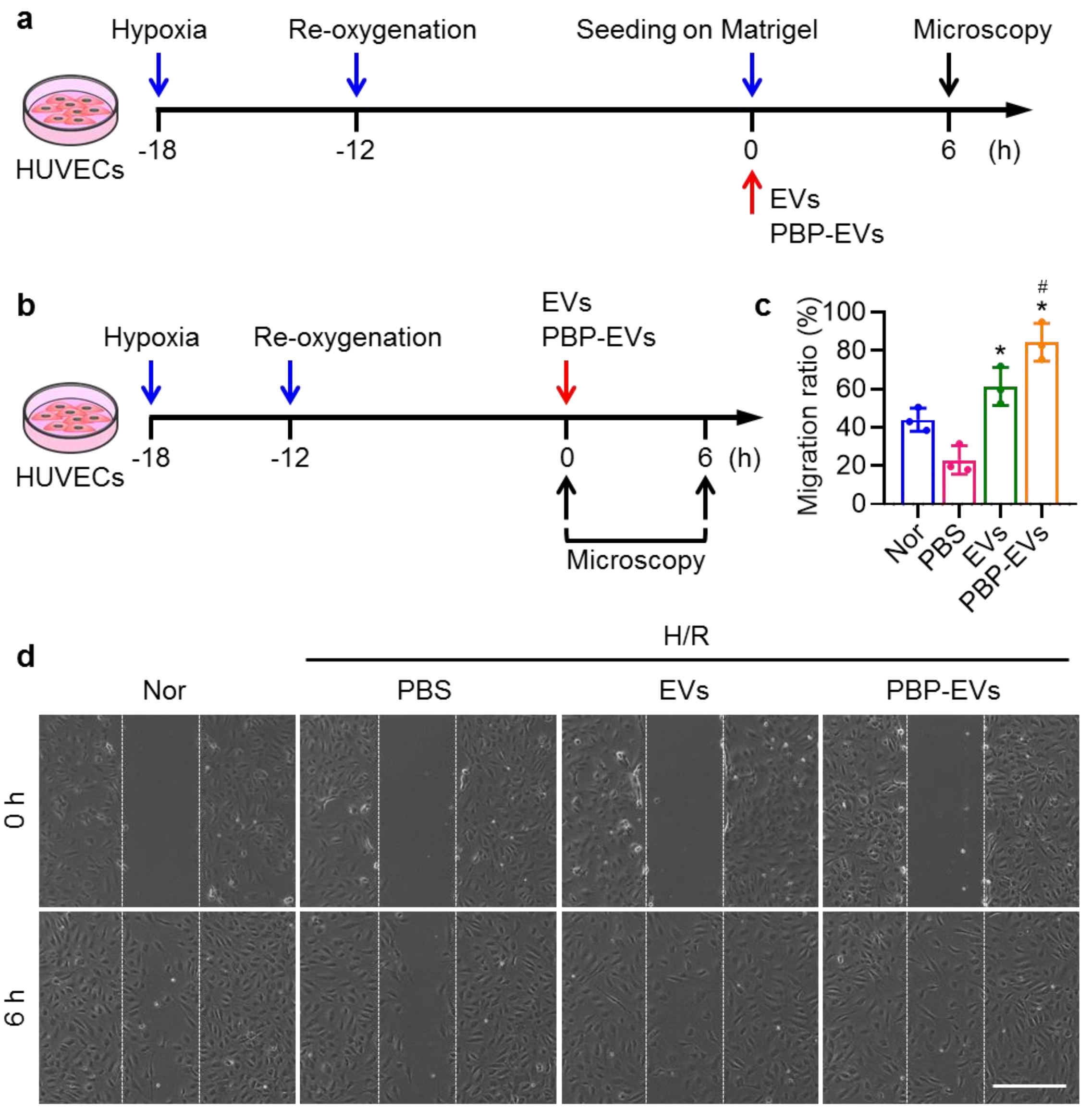

Supplementary Fig. 14 | PBP-EVs ameliorated the angiogenic capacity of H/R injured HUVECs. a, Schematic diagram of the study design for the tube formation assay. **b**, Schematic diagram of study design for wound healing assay. **c**, Migration ratio of HUVECs in the wound healing assay. n = 3. All data are expressed as mean ± s.d. Statistical analysis was performed using one-way ANOVA with Tukey's multiple comparison tests. **P* < 0.05 versus PBS, #*P* < 0.05 versus EVs. **d**, Representative images of the wound healing assay. Scale bar, 100 μm.

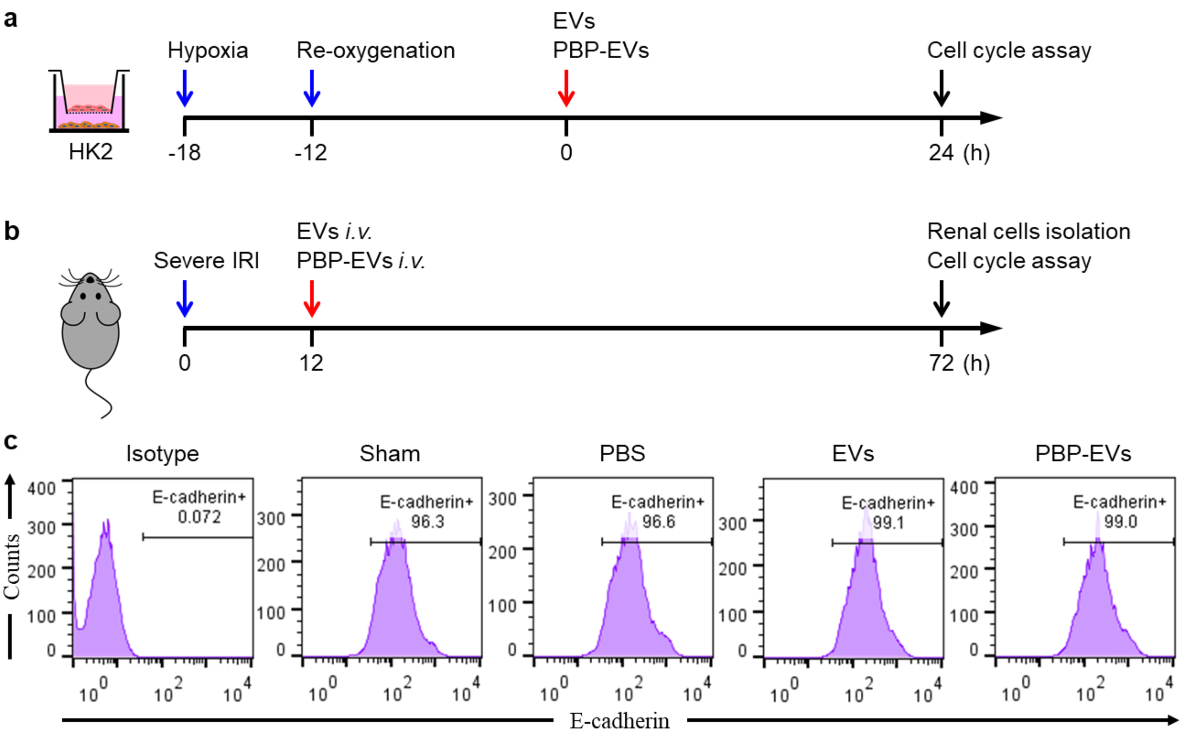

Supplementary Fig. 15 | Cell cycle assays of tubular epithelial cells *in vivo* and *vitro*. a, Schematic diagram of study design for the cell cycle assay of H/R injured HK2. **b**, Schematic diagram of study design for isolation of single cells from injured kidneys for cell cycle assay. **c**, Quantitative analysis of E-cadherin expression in cells isolated from the injured kidney by flow cytometry.

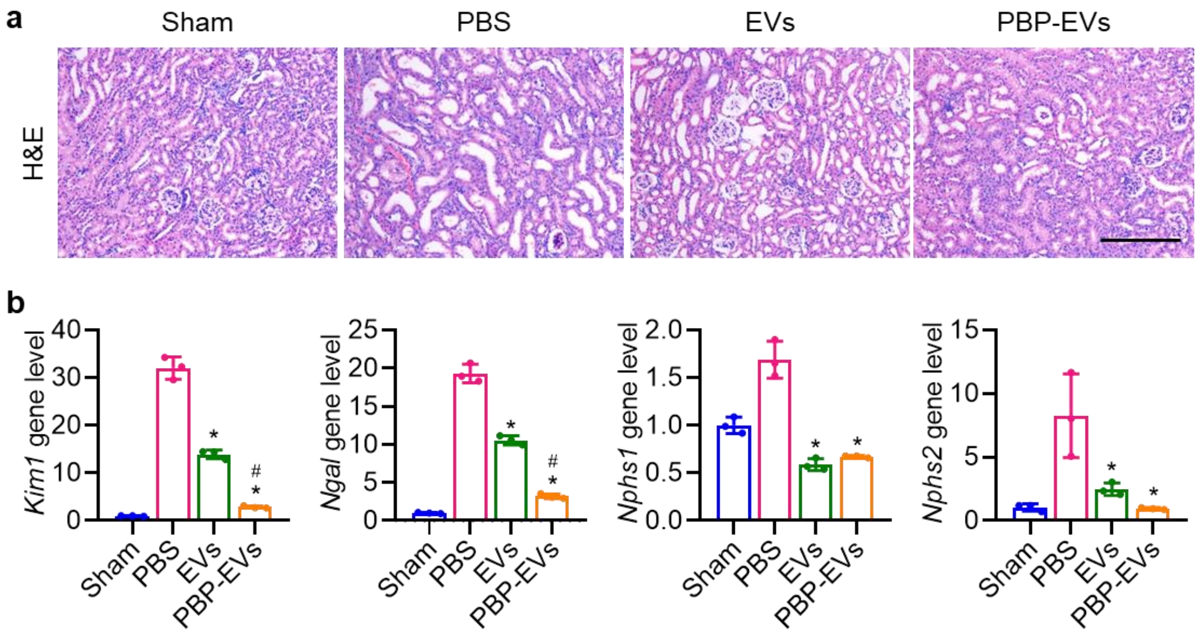

Supplementary Fig. 16 | PBP-EVs attenuated renal injury. a, H&E staining of renal tissues 3 days post severe IRI. Scale bar, 200 μm. **b**, Expressions of genes associated with kidney injury (*Kim1*, *Ngal*, *Nphs1*, and *Nphs2*) in renal tissues were detected by real-time qPCR on day 3 post severe IRI. The renal tissues with the sham operation served as control. n = 3. All data are expressed as mean ± s.d. statistical analysis was performed using one-way ANOVA with Tukey's multiple comparison tests. *$P < 0.05$ versus PBS, #$P < 0.05$ versus EVs.

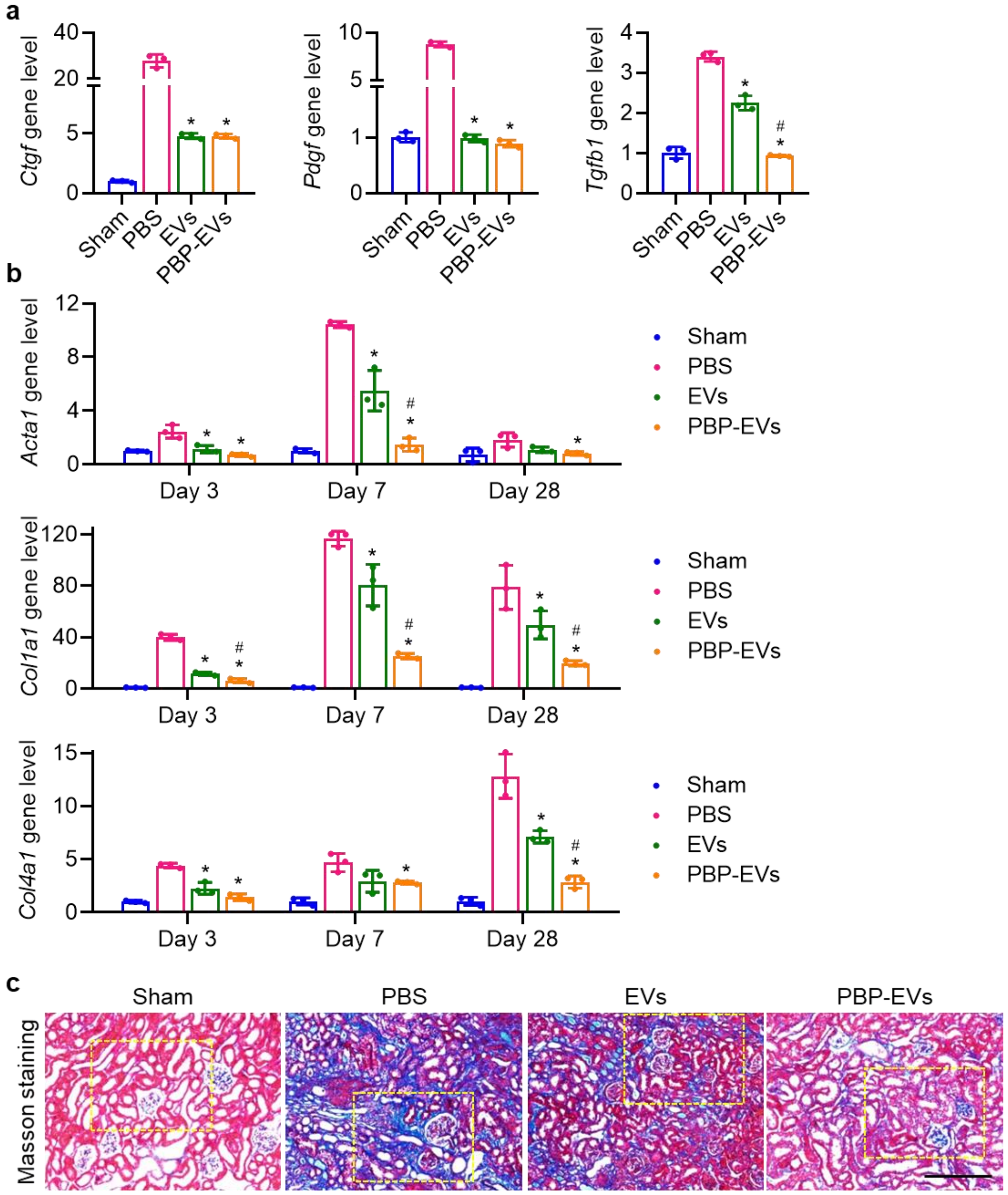

Supplementary Fig. 17 | PBP-EVs attenuated renal fibrosis. **a**, Expression of profibrogenic genes (*Ctgf*, *Pdgf*, and *Tgfb1*) in renal tissues was detected by real-time qPCR on day 3 post severe IRI. n = 3. **b**, The expressions of fibrosis-associated genes (*Acta1*, *Col1a1*, and *Col4a1*) in renal tissues were detected by real-time qPCR at 3, 7, and 28 days post severe IRI. n = 3. **c**, Masson's trichrome staining of renal tissues 28 days post severe IRI. The magnified zones showed in Fig 7e were indicated in the *yellow* boxes. Scale bar, 200 μm. For **a** statistical analysis was performed using one-way ANOVA with Tukey's multiple comparison tests. For **b**, statistical analysis was performed using two-way ANOVA with Tukey's multiple comparison tests. $^{*}P < 0.05$ versus PBS, $^{\#}P < 0.05$ versus EVs.

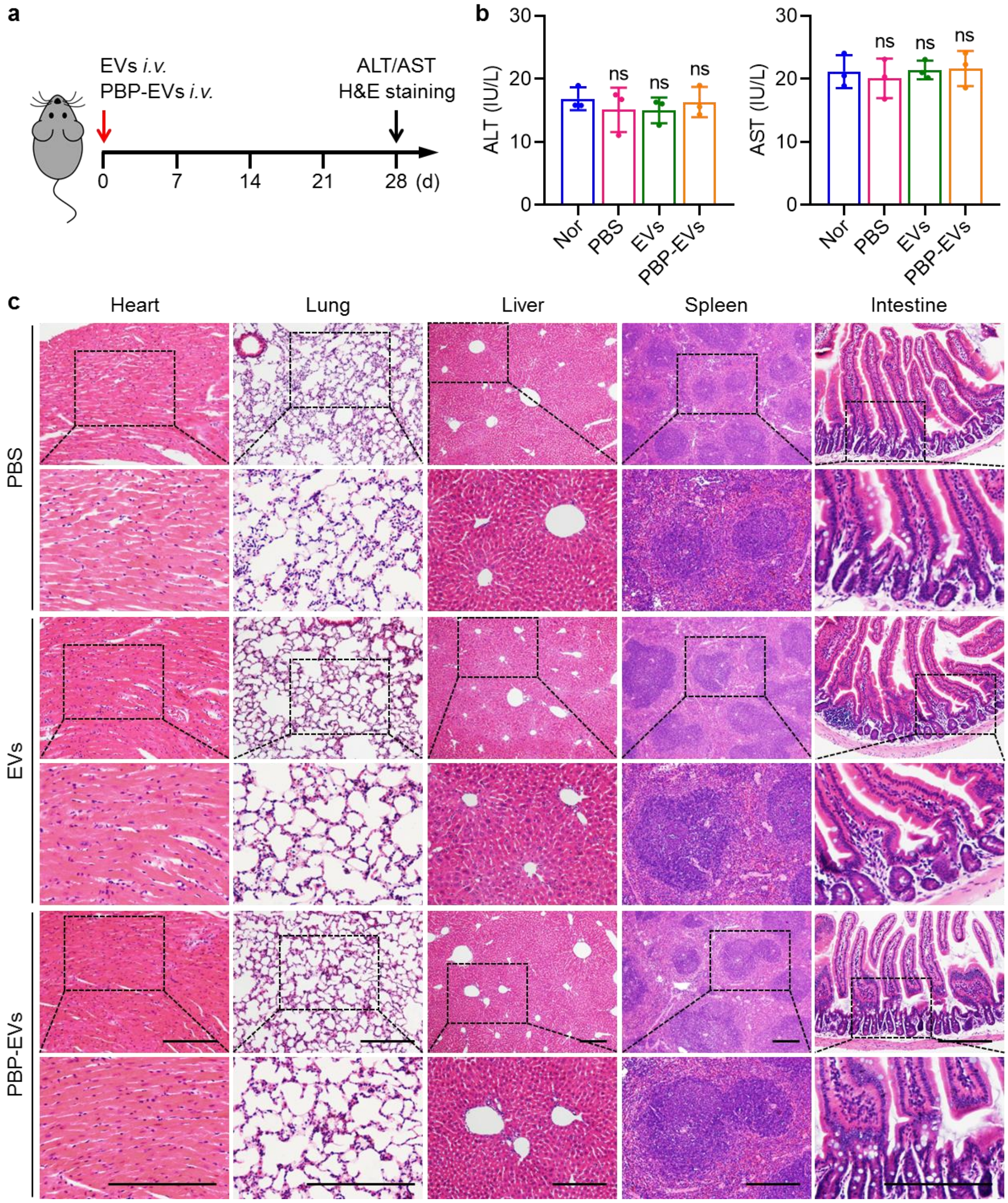

Supplementary Fig. 18 | Safety verification of PBP-EVs. a, Schematic diagram of study design for safety verification. **b**, Serum levels of alanine transaminase (ALT) and aspartate transaminase (AST) were tested at day 28 post injection of PBP-EVs. n = 3. All data are expressed as mean ± s.d. Statistical analysis was performed using one-way ANOVA with Tukey's multiple comparison tests. ns, not significant versus Nor. **c**, H&E staining of the major organs (heart, lung, liver, spleen, and intestine) at 28 days post injection of PBP-EVs. Scale bar, 200 μm.

a

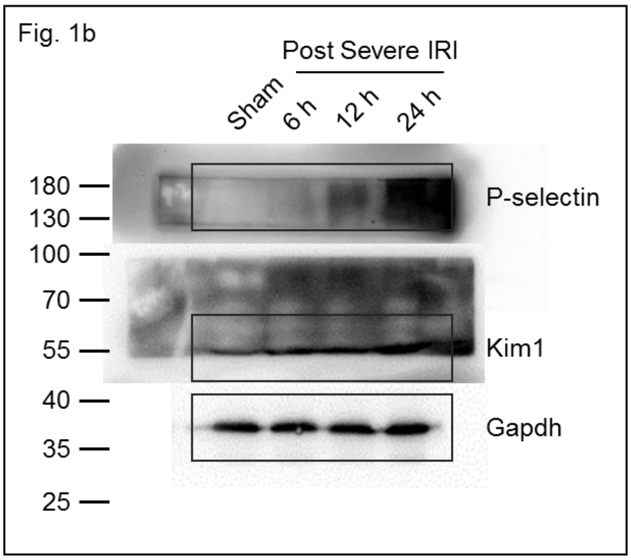

b

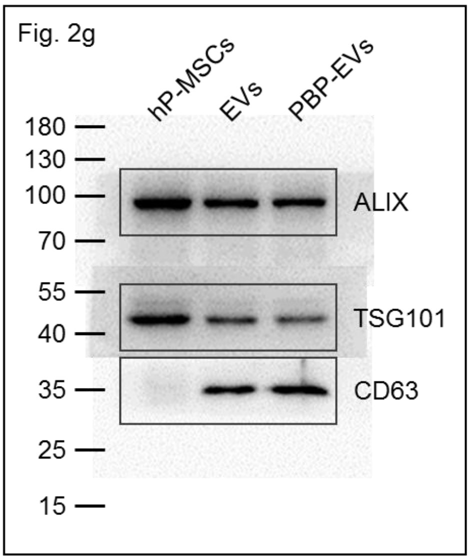

Supplementary Fig. 19 | Full scans of western blots. a, Full scans of western blot bands in Fig. 1b. **b,** Full scans of Western blot bands in Fig. 2g.

Supplementary Table. 1 | Primers used in the real-time qPCR assay.

Gene	Forward primer sequence, 5’-3’	Reverse primer sequence, 5’-3’
Selp	CCTCCCGAATGTCAAGCTGT	CTCTGGGGCGAAGATTCCTG
Sele	GCTACCCATGGAACACGACA	CTTTGCATGATGGCGTCTCG
Icam1	TTCTTTTGCTCTGCCGCTCT	CCTCTTGCCAGGTCCAGTTC
Vcam1	CTGGGAAGCTGGAACGAAGT	GCCAAACACTTGACCGTGAC
Pecam1	CCACCTCGAAAAGCAGGTCT	TTGGATACGCCATGCACCTT
Vegfa	CCCACGTCAGAGAGCAACAT	CCGGGATTTCTTGCGCTTTC
Vegfr2(Kdr)	TGGGCAGTCAAGTCCGAATC	GTTGGTGAGGATGACCGTGT
Ang1(Angpt1)	TGGGGGAGGTTGGACAGTAA	CTGTGAGTAGGCTCGGTTCC
Ang2	AAAGGAAGCCCTTATGGACGAA	CCAGCCATTCTCACAGCCAAT
Plgf(Pgf)	TTCTGAGTCGCTGTAGTGGC	TGCCTTTGTCGTCTCCAGAA
Egf	CTGTTGTTGGAGGGAGCGAT	CGTGATTCCACTGGGTTCCA
Fas	TTGCTGGCTCACAGTTAAGA	TTCAGGTTGGCATGGTTGAC
Fasl	GGTCAGTTTTTCCCTGTCCA	AATCCCATTCCAACCAGAGC
Bax	CATGTTTGCTGATGGCAACT	TGATCAGCTCGGGCACTTTA
Bad	GAGTGAGCAGGAAGACGCTA	CCCTGCTGATGAATGTTGCT
Ctgf(Ccn2)	CCCAACTATGATGCGAGCCA	CGGATGCACTTTTTGCCCTT
Pdgfb	TCCGTAGATGAAGATGGGGC	TGGTGCGATCGATGAGGTTC
Tgfb1	ACAATTCCTGGCGTTACCTT	CCCTGTATTCCGTCTCCTTG
Kim1(Havcr1)	ACCCACTACATTTTGTCCCCA	GTGCCATTCCAGTCTGTAGGA
Ngal(Lcn2)	GGAACGTTTCACCCGCTTTG	CATTGGTCGGTGGGGACAGA
Nphs1	TATCGCCAAGCCTTCACAGG	CAGCGAAGGTCATAGGGTC
Nphs2	GTAGTGGACGTGGACGAGGTTC	CTTGATGCCTTCCTCTGGTCG
Acta1	TCTTGTGTGTGACAACGGCT	GGGTCAGGATACCTCGCTTG
Col1a1	CCAATGGTGAGACGTGGAA	GTCCCTCGACTCCTACATCT
Col4a1	TGGGTCCTCCGGGATTTACT	TCTCCTGTTGGGGCAAAGTC
Gapdh	GAGTGTTTCCTCGTCCCGTAG	CAATCTCCACTTTGCCACTGC

Supplementary Methods

MicroRNA profiling.

Total RNAs were extracted from 1 mg of EVs using TRIzol reagent (Invitrogen) as the manufacturer's instruction. Subsequently, the total RNA samples were submitted to the Sangon Biotech (Shanghai, China) Co., Ltd. for library preparation and sequencing. In brief, 2 μg total RNA per sample was prepared for miRNA sequencing libraries generation by using NEBNext® Multiplex Small RNA Library Prep Set for Illumina® (NEB, USA) following the manufacturer's recommendations. The library quality was assessed on the Agilent Bioanalyzer 2100 system and sequenced on an Illumina HiSeq X-ten platform. Data analysis was performed by Sangon Biotech (Shanghai) Co., Ltd.

P-selectin binding peptide (PBP) and DMPE-PEG-PBP conjugates.

The P-selectin-binding peptide (PBP; DAEWVDVS) with purity >99% was synthesized by GL Biochem (Shanghai, China). To modify the EVs with the PBP, the DMPE-PEG-PBP (DPP) conjugates were synthesized as previously reported[1]. The N-terminus of PBP has added a cysteine (C) to produce a thiol at the N-terminus, which could subsequently react with the amphipathic material DMPE-PEG5000-Maleimide (DMPE-PEG-Mal; MW 5 kDa, Ponsure Biotechnology, Shanghai, China). Then, 30 mg of DMPE-PEG-MAL and 30 mg of PBP were dissolved in 2 mL of dimethylformamide, followed by adjustment of pH to 8.0 using triethylamine. After stirring for 24 h at room temperature under N_2 atmosphere, the reaction mixture was dialyzed against deionized water using a dialysis membrane (MWCO 3,000 Da) for three days. The reaction product was lyophilized to obtain the DPP powder.

The chemical structure of the DPP conjugates was characterized by NMR (1H NMR, Mercury Vx-300), using D_2O as the solvent.

The fluorescent dye Cyanine5.5 (Cy5.5) was conjugated to the amino group of cysteine to visualize PBP *in vitro* and *vivo*. Cy5.5 labeled DPP conjugates were synthesized by adding an excess amount of Cy5.5 NHS ester (10-fold molar excess, 27020, Lumiprobe Corporation, Hunt Valley, MD) to DPP aqueous solution with stirring at 4 °C for 24 h under dark conditions. The reaction mixture was dialyzed against deionized water with a dialysis membrane for 3 days and then lyophilized to obtain the Cy5.5-labeled DPP powder. The conjugates of DMPE-PEG-Cy5.5 were used as control were synthesized under the same reaction conditions.

Cell targeting and internalization of PBP-EVs.

HUVECs (5×10^4 cells per well) were cultured on glass coverslips in a 24-well plate 1 day prior to H/R administration. Normal HUVECs served as control. Then, Cy5.5/Gluc-labeled EVs and PBP-EVs were added to the medium of HUVECs incubating for another 2 h. After washing by BPS twice, the internalization of EVs and PBP-EVs was first detected in the presence of the CTZ using the IVIS Lumina Imaging System. Subsequently, the internalized EVs and PBP-EVs in HUVECs were visualized by Cy5.5. HUVECs were fixed and counterstained with Alexa Fluor 488 labeled phalloidin (1:400; Invitrogen, Carlsbad, CA) and 4′,6-diamidino-2-phenylindole (DAPI, 1:2,000; Vector Laboratories, Burlingame, CA). The images were captured under an LSCM and analyzed by using ImageJ software.

Penetration of PBP-EVs through endothelial barriers

The penetration of EVs and PBP-EVs through a monolayer of endothelial cells was evaluated using a modified Transwell system (3472, Corning, Corning, NY) as previously described[2]. HUVECs (1×10^4 cells per well) were seeded in the upper chambers of Transwell inserted into 24-well plates and cultured for an additional 7 days. The medium was replaced every other day. The confluence of the monolayer was checked to ensure that the next steps were conducted in the absence of gaps in cell junctions. Subsequently, HK2 (5×10^4 cells per well) were cultured on glass coverslips in the lower chambers (24-well plate) and the upper chambers with the HUVEC monolayer were inserted. After the Transwell systems were performed H/R administration, the Cy5.5/Gluc-labeled EVs or PBP-EVs (100 μg/mL) were added into the upper chambers and incubated for another 6 h. The media of the upper and lower chambers were collected and measured in the presence of CTZ by Gluc imaging. HK2 cultured in the lower chambers were fixed and counterstained with Alexa Fluor 488-labeled phalloidin and DAPI for LSCM analysis.

Blocking effect of PBP-EVs

The adhesion of monocytes to injured endothelial cells was examined to assess the blocking effect of PBP-EVs on P-selectin. The normal or H/R administrated HUVECs (2×10^4 cells per well) were cultured on glass coverslips in 48-well plates and co-incubated with human monocyte THP-1 (5×10^3 cells per well). EVs or PBP-EVs (100 μg/mL) were added to one of the co-culture systems respectively and shaking incubated at 37 °C for 3 h. After removing

unbound THP-1 with PBS, the cells on glass coverslips were fixed with 2.5% glutaraldehyde and dehydrated with serial dilutions of ethanol. The morphology and amounts of THP-1 bound to HUVECs were observed and analyzed by scanning electron microscopy (SEM; Phenom, Shanghai, China).

Myeloperoxidase (MPO) assay of renal tissues.

The MPO activities of renal tissues were measured using the MPO assay kit (Nanjing Jiancheng Bioengineering Institute, Nanjing, China). On day 3 post severe IRI, a quarter of the kidneys were homogenized to obtain 5% homogenate according to the manufacturer's instructions. The homogenates were centrifuged at 5,000 g for 5 min and the supernatants were removed for the MPO assay.

Endothelial cell function analysis

The tube formation assay and the wound healing assay were performed to analyze endothelial cell functions as previously reported[3]. Briefly, normal or H/R administrated HUVECs used for tube formation were seeded in growth factor-reduced Matrigel (Corning) coated 48-well plates and treated with EVs or PBP-EVs (100 μg/mL) for 6 h. The images of network structures were captured by a bright-field microscope (Olympus) and analyzed by ImageJ software to evaluate the proangiogenic capacity of HUVECs. Regarding the wound healing assay, a scratch wound was generated in the monolayer of normal or H/R administrated HUVECs and cultured for an additional 12 h with 100 μg/mL EVs or PBP-EVs. The images of wounds captured at 0 and 12 h were used to quantify the migration ratio using ImageJ

software.

Real-time quantitative polymerase chain reaction (qPCR).

Total RNA samples were extracted from renal tissues with TRIzol reagent following the manufacturer's protocol. Subsequently, 2 μg total RNAs were reversely transcribed into cDNA using BioScript All-in-One cDNA Synthesis SuperMix (Bimake, Houston, TX). Real-time qPCR analysis of relative gene expression was carried out using qPCR SYBR green master mix (Yeasen, Shanghai, China) on a CFX96 Touch System (Bio-Rad, Hercules, CA). The primer sequences (listed in Supplementary Table. 1) were designed using the NCBI *Primer-BLAST* tool (http://www.ncbi.nlm.nih.gov/tools/primer-blast/) and were synthesized by Sangon Biotech (Beijing) Co., Ltd. Relative gene expression folding changes were identified with the $2^{-\Delta\Delta Ct}$ method and normalized to *Gapdh*.

Western blot analysis.

Renal tissue and EV lysates in the radioimmunoprecipitation assay (RIPA; Solarbio, Beijing, China) buffer were subjected to Western blotting analysis. Proteins were heat-denaturized in 5× SDS-PAGE loading buffer (CWBIO, Beijing, China), fractionated in 10% SDS-polyacrylamide gels, electro-transferred onto polyvinylidene fluoride (PVDF) membranes (Millipore, Darmstadt, Germany) and immunoblotted with the indicated antibodies. The following antibodies were used according to the manufacturer's instructions: P-selectin (1:100; sc-8419, Santa Cruz Biotechnology, Santa Cruz, CA), Kim1 (1:1,000; ab47635, Abcam, Cambridge, MA), Gapdh (1:10,000; 60004-1-Ig, Proteintech, Chicago, IL),

ALIX (1:2,000; WL03063, Wanleibio, Shengyang, China), TSG101(1:2,000; ET1071-59, HuaAn Biotechnology, Hangzhou, China) and CD63 (1:2,000; WL02549, Wanleibio). The blot bands were visualized with enhanced chemiluminescence detection reagent (Thermo Scientific) and analyzed by ImageJ software. Full scans of all western blots are supplied in **Supplementary Fig. 19**.

Systematic toxicity evaluation.

To assess the systematic toxicity of EVs and PBP-EVs, male C57BL/6 mice (n = 3) were injected intravenously with EVs or DPP-EVs (100 μg at a total volume of 200 μL per mouse). Mice injected with 200 μL PBS were used as control. On day 28 post injection, the serum samples were collected for alanine transaminase (ALT) and aspartate aminotransferase (AST) tests. ALT and AST were evaluated using the ALT assay kit (Nanjing Jiancheng Bioengineering Institute) and the AST assay kit (Nanjing Jiancheng Bioengineering Institute), respectively. The major organs (including the heart, lung, liver, spleen, and intestine) were harvested and fixed in paraformaldehyde for further H&E staining.

References

1. Yan, H. et al. Targeted repair of vascular injury by adipose-derived stem cells modified with P-selectin binding peptide. *Adv. Sci.* **7**, 1903516 (2020).
2. Shamay, Y. et al. P-selectin is a nanotherapeutic delivery target in the tumor microenvironment. *Sci. Transl. Med.* **8**, 345ra387 (2016).
3. Du, W. et al. Enhanced proangiogenic potential of mesenchymal stem cell-derived exosomes stimulated by a nitric oxide releasing polymer. *Biomaterials* **133**, 70-81 (2017).
